# Noncovalent microarrays from synthetic amino-terminating glycans: Implications in expanding glycan microarray diversity and platform comparison

**DOI:** 10.1093/glycob/cwab037

**Published:** 2021-05-08

**Authors:** Chunxia Li, Angelina S Palma, Pengtao Zhang, Yibing Zhang, Chao Gao, Lisete M Silva, Zhen Li, Filipa Trovão, Markus Weishaupt, Peter H Seeberger, Leonid M Likhosherstov, Vladimir Piskarev, Jin Yu, Ulrika Westerlind, Wengang Chai

**Affiliations:** Key Laboratory of Marine Drugs, Ministry of Education, School of Medicine and Pharmacy and Shandong Provincial Key laboratory of Glycoscience and Glycoengineering, Ocean University of China, 5 Yushan Road, Qingdao 266003, China; Laboratory for Marine Drugs and Bioproducts, Pilot National Laboratory for Marine Science and Technology (Qingdao), Wenhai Road, Qingdao 266237, China; Applied Molecular Biosciences Unit, Department of Chemistry, School of Science and Technology, NOVA University of Lisbon, Campus de, 1099-085 Lisboa, Portugal; Key Laboratory of Marine Drugs, Ministry of Education, School of Medicine and Pharmacy and Shandong Provincial Key laboratory of Glycoscience and Glycoengineering, Ocean University of China, 5 Yushan Road, Qingdao 266003, China; Glycosciences Laboratory, Faculty of Medicine, Imperial College London, Du Cane Road, London W12 0NN, United Kingdom; Glycosciences Laboratory, Faculty of Medicine, Imperial College London, Du Cane Road, London W12 0NN, United Kingdom; Glycosciences Laboratory, Faculty of Medicine, Imperial College London, Du Cane Road, London W12 0NN, United Kingdom; Glycosciences Laboratory, Faculty of Medicine, Imperial College London, Du Cane Road, London W12 0NN, United Kingdom; Applied Molecular Biosciences Unit, Department of Chemistry, School of Science and Technology, NOVA University of Lisbon, Campus de, 1099-085 Lisboa, Portugal; Department of Biomolecular Systems, Max-Planck-Institute of Colloids and Interfaces, Am Mühlenberg 1, 14476 Potsdam, Germany; Department of Biomolecular Systems, Max-Planck-Institute of Colloids and Interfaces, Am Mühlenberg 1, 14476 Potsdam, Germany; N.D. Zelinsky Institute of Organic Chemistry, Russian Academy of Sciences, Leninskiy Prospekt 47, Moscow 119334, Russia; Nesmeyanov Institute of Organoelement Compounds, Russian Academy of Sciences, Vavilova St. 28, Moscow V-334, 119991, Russia; Umeå University, Department of Chemistry, KBC-building, Linneaus väg 6, S-907 36 Umeå, Sweden; Umeå University, Department of Chemistry, KBC-building, Linneaus väg 6, S-907 36 Umeå, Sweden; Glycosciences Laboratory, Faculty of Medicine, Imperial College London, Du Cane Road, London W12 0NN, United Kingdom

**Keywords:** Dectin-1, glycan micorarray, oligosaccharide microarray, neoglycolipid, rotavirus

## Abstract

Glycan microarrays have played important roles in detection and specificity assignment of glycan recognition by proteins. However, the size and diversity of glycan libraries in current microarray systems are small compared to estimated glycomes, and these may lead to missed detection or incomplete assignment. For microarray construction, covalent and noncovalent immobilization are the two types of methods used, but a direct comparison of results from the two platforms is required. Here we develop a chemical strategy to prepare lipid-linked probes from both naturally derived aldehyde-terminating and synthetic amino-terminating glycans that addresses the two aspects: expansion of sequence-defined glycan libraries and comparison of the two platforms. We demonstrate the specific recognition by plant and mammalian lectins, carbohydrate-binding modules and antibodies and the overall similarities from the two platforms. Our results provide new knowledge on unique glycan-binding specificities for the immune receptor Dectin-1 toward β-glucans and the interaction of rotavirus P[19] adhesive protein with mucin *O*-glycan cores.

## Introduction

Recognition of glycans by proteins is crucial to understand molecular mechanisms in health and disease. Carbohydrate microarrays, including those of polysaccharides (Wang et al. 2002) and sequence-defined glycans (Fukui et al. 2002), have played a major role in dissecting glycan–protein interactions after their emergence as a natural follow-up to the development of the microarray technologies for nucleic acids (Schena and Shalon 1995) and proteins (MacBeath and Schreiber 2000). Since their inception in 2002, glycan microarrays have proven to be powerful tools in the detection and specificity assignment of glycan–protein interactions with implications in biology and medicine.

Natural glycans cannot be arrayed directly due mainly to their highly hydrophilic nature and the incompatibility of the functional groups of carbohydrate molecules with readily available microarray slides. Various approaches were developed to convert glycans into forms suitable for printing and immobilization on different surface-modified glass slides used for arrays of nucleic acids and proteins. As carbohydrate molecules cannot be cloned, their isolation from natural glycome sources (Song et al. 2011; Palma et al. 2015; Li et al. 2018) or synthesis by chemical (Cheng et al. 2018; Geissner et al. 2019) and enzymatic means (Prudden et al. 2017; Gao et al. 2019) are the main methods for building up libraries of glycans. Many microarray platforms using sequence-defined glycans have been developed using different chemistries and immobilization strategies to address specific biological questions or to target specific glycomes (Fukui et al. 2002; Blixt et al. 2004; Park et al. 2007; Shipp and Hsieh-Wilson 2007; Ban and Mrksich 2008; Wang et al. 2009; Sanchez-Ruiz et al. 2011; Šardzík et al. 2011; Pedersen et al. 2012; Xia and Gildersleeve 2015; Geissner et al. 2019). Among these, the neoglycolipid (NGL)-based microarray system of the Imperial College Glycosciences Laboratory (Fukui et al. 2002; Palma et al. 2014), the platform of US Consortium for Functional Glycomics (CFG) (Blixt et al. 2004) and the microbe-focused Max Planck Institute (MPI) platform (Geissner et al. 2019) have glycan libraries in a scale and diversity suitable for broad screening analyses and are major international resources serving the wider scientific community. The CFG and MPI arrays comprise amino-terminating synthetic glycans that are covalently immobilized on N-hydroxysuccinimide (NHS)-functionalized slides, whereas the NGL arrays comprise mainly naturally derived aldehyde (in the form of hemiacetal)-terminating glycans conjugated to a long chain amino-phospholipid and noncovalently immobilized on nitrocellulose-coated slides. The three platforms are in some ways complementary with partial overlap in their glycan repertoires.

Although debatable, it has been estimated that there are 100,000–500,000 glycan structures in the mammalian glycome (Freeze 2006; Rillahan and Paulson 2011) present on glycoproteins, glycolipids and polysaccharides and as secreted free sugars. The numbers of peripheral sequences (Drickamer and Taylor 2002) or glycan determinants (Cummings 2009) are in the range of 7000. Therefore, the glycome is considered larger than the genome and proteome, but the size of mammalian glycan array libraries up to now is small compared with those assembled for DNA and protein microarrays (Zhu et al. 2001). Currently in each of the two largest glycan microarrays there are around 1000 probes; some of which have the same glycan structures but with different linkers or tags. There is an obvious need to expand the libraries of sequence-defined glycan probes to cover the major part of glycan structures within glycomes.

Given the different ways of constructing glycan microarrays and the vast diversity of carbohydrate molecules with different structural and chemical/physical properties over nucleic acids and proteins, careful comparisons across different platforms are necessary for widening the scope and future use of microarrays in diverse applications and in deriving glycan-binding specificities with confidence. In light of this, there have been studies comparing glycan-binding profiles obtained with microarrays that use different chemistries for glycan derivatization, glycan linker types, glycan probe densities and modes of presentation (Padler-Karavani et al. 2012; Grant et al. 2014; Wang et al. 2014; Temme et al. 2019). However, a focused study on comparison of the two of the major platforms, covalent and noncovalent, has not been carried out.

**Scheme 1:**
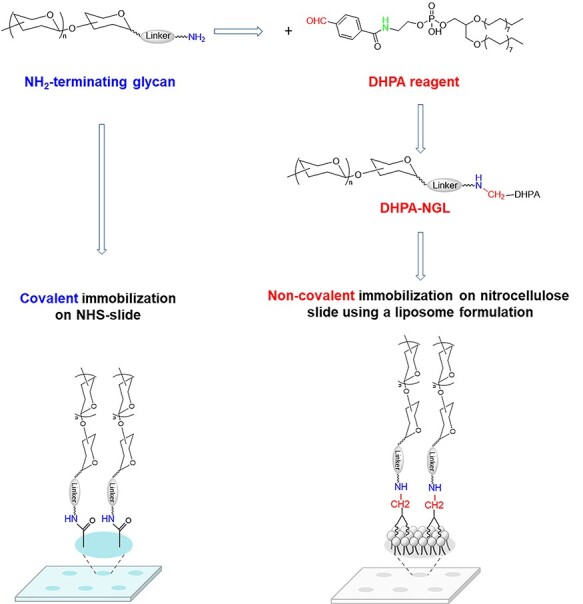
Amino-terminating glycans for both covalent and noncovalent arraying.

The present work has aimed to address the two aspects: the need to expand libraries of sequence-defined glycans and for comparison of two of the major microarray platforms, by developing a new lipid reagent, which is suitable for preparation of NGL probes from amino-terminating glycans. This opens the way for NGL microarrays to be sourced from both naturally derived aldehyde-terminating and synthetic amino-terminating glycans, and therefore expansion of the microarray coverage. It is now also possible to compare the two platforms using the very same amino-terminating glycans as probes for both covalent and noncovalent microarrays after their conversion into NGLs (Scheme 1). Although the method presented is only applicable to the NGL noncovalent array platform, the conclusions from the specifically designed and conducted comparison of the two most widely used platforms employed by the international resources and from the probe construction strategy using both naturally derived aldehyde- and synthetic amino-terminating glycans to increase size and the diversity of probe libraries should have wider implications to the microarray users and providers.

## Results

### Synthesis of novel phospholipid reagents active for amino-terminating glycans

Currently, the amino-phospholipid 1,2-dihexadecyl-*sn*-glycero-3-phosphoethanolamine (DHPE) is used to conjugate aldehyde-terminating glycans by reductive amination to form NGLs (Chai et al. 2003). Here we attempted two strategies to convert DHPE into lipid reagents with functionalities reactive with amino-terminating glycans.

**Fig. 1:**
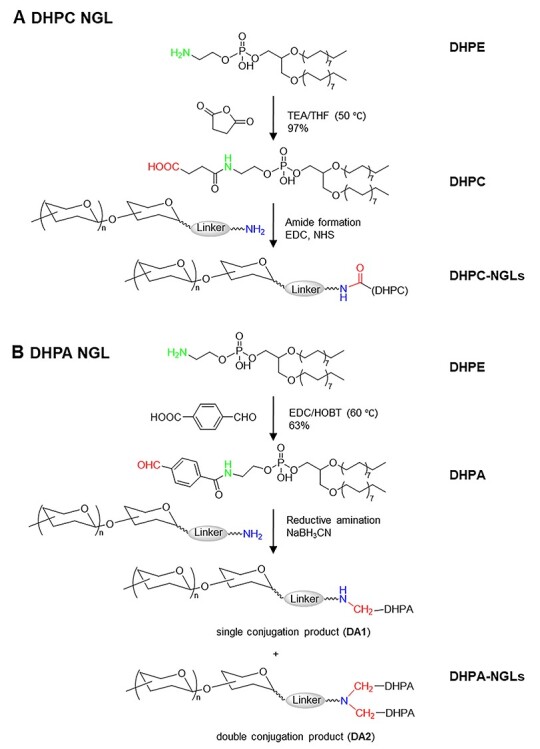
Reaction schemes of novel lipid reagents for NGL preparation from amino-terminating sugars.

A carboxyl-terminating lipid was designed for amide condensation with amino-terminating sugars. The amino group of DHPE reacted with succinic anhydride to form *N*-(4-oxobutanoic acid)-DHPE (DHPC) (Figure 1A), which contains a terminal carboxyl to be used for conjugation with amino-terminating sugars. The new lipid reagent DHPC was obtained in good yield (97%).

An aldehyde-terminating lipid was also designed by reaction of DHPE and hetero-bifunctional 4-carboxybenzaldehyde (Figure 1B). The carboxyl was used for conjugation to the amino group of DHPE by formation of an amide bond with the aid of activation by 1-ethyl-3-(3-dimethylaminopropyl)carbodiimide (EDC) and *N*-hydroxybenzotriazole (HOBT), leaving the free aldehyde for linking to amino-terminating glycans by reductive amination. The aldehyde-terminating lipid product *N*-(4-formylbenzamide)-DHPE (DHPA) was obtained only in a moderate yield (63%) due mainly to the poor solubility of DHPE in the reaction solvent (dichloromethane or chloroform).

For assessing the use of the two lipid reagents in preparation of NGLs the aminoethyl glycoside of galactose, Galβ-O-CH_2_CH_2_-NH_2_ (abbreviated as Gal-C2-NH_2_), was used as the model sugar.

DHPC conjugation with Gal-C2-NH_2_ via amide condensation was carried out in the presence of the activation reagents EDC and NHS (Figure 1A) (Sam et al. 2010). However, as indicated by high-performance thin layer chromatography (HPTLC), only a limited amount NGL was generated (Figure S1A).

DHPA was conjugated to Gal-C2-NH_2_ via reductive amination (Figure 1B). The amino group of the amino-terminating sugar was linked to the aldehyde of DHPA in the presence of reducing agent cyanoborohydride. HPTLC analysis showed that Gal-C2-NH_2_ was almost completely converted into NGL (Figure S1B and Figure S2A). Due to the higher yield (85%, Figure S1B and Figure S2A) of NGL products and the potential use of the UV chromophore afforded by the benzene ring, DHPA was selected for preparation of a library of NGL probes.

### Preparation of DA-NGLs for exploratory noncovalent microarray construction and analysis

**Fig. 2:**
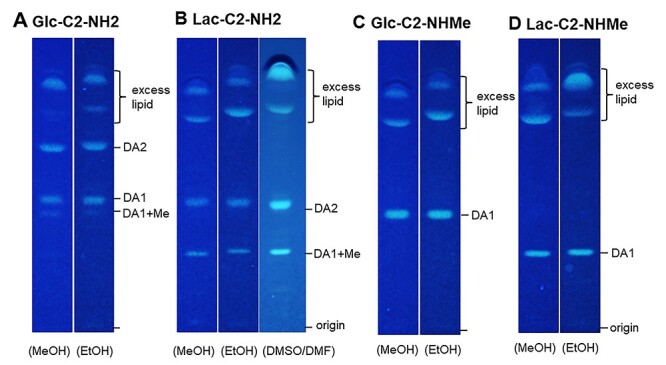
HPTLC analysis of DA-NGL products. (**A**) Multiple products formed from Glc-C2-NH2. (**B**) Reaction products from Lac-C2-NH2 using MeOH, EtOH, DMSO and DMF as the solvent. (**C**) Reaction products from methylamino-terminating Glc-C2-NHMe and Lac-C2-NHMe using MeOH and EtOH as the solvent. DA1, single lipid conjugation product; DA2, double lipid conjugation product; DA1 + Me methylated single lipid conjugation product.

Analyses by HPTLC and MS indicated that multiple NGL products can be formed with DHPA. In the case of Glc-C2-NH_2_, three DHPA-NGL products were found: NGL with single lipid (DA1), two lipids (DA2) and methylated single lipid (DA1 + Me), as revealed by HPTLC (Figure 2A) and MALDI-MS (Figure S2B). The double lipid conjugation by reductive amination with the secondary amine was unpredicted. The formation of DA2-NGLs could not be minimized under different conjugation conditions (see Methods section for details). Aiming to minimize the formation of methylated products, EtOH, DMSO or DMF were used instead of MeOH, but methylated product was still formed (Figure 2B).

To obtain products with a single lipid, methylamino-terminating sugars were used. In this case, a clean single product was obtained (Figure 2C and D). An aminooxy-functionalized glycan, GalNAcα1-ONH_2_ can also be conjugated to DHPA by generating an oxime without reduction to give a single lipid-linked DA-NGL (Figure S3).

HPTLC analysis showed that the yield of these products (including NGLs with single and double lipid and methylated) was greater than 80% after 6–24 h for most of the oligosaccharides used in this work (Table SI). Conjugation was less efficient for aromatic amine-functionalized heparin-derived glycans, Hep-4-NS-PhNH_2_ and Hep-4-NAc-PhNH_2_. For these two oligosaccharides, incubation was prolonged to 48 h and at a higher temperature (80°C). The conjugation efficiency was thereby increased to ~50% (data not shown).

**Fig. 3:**
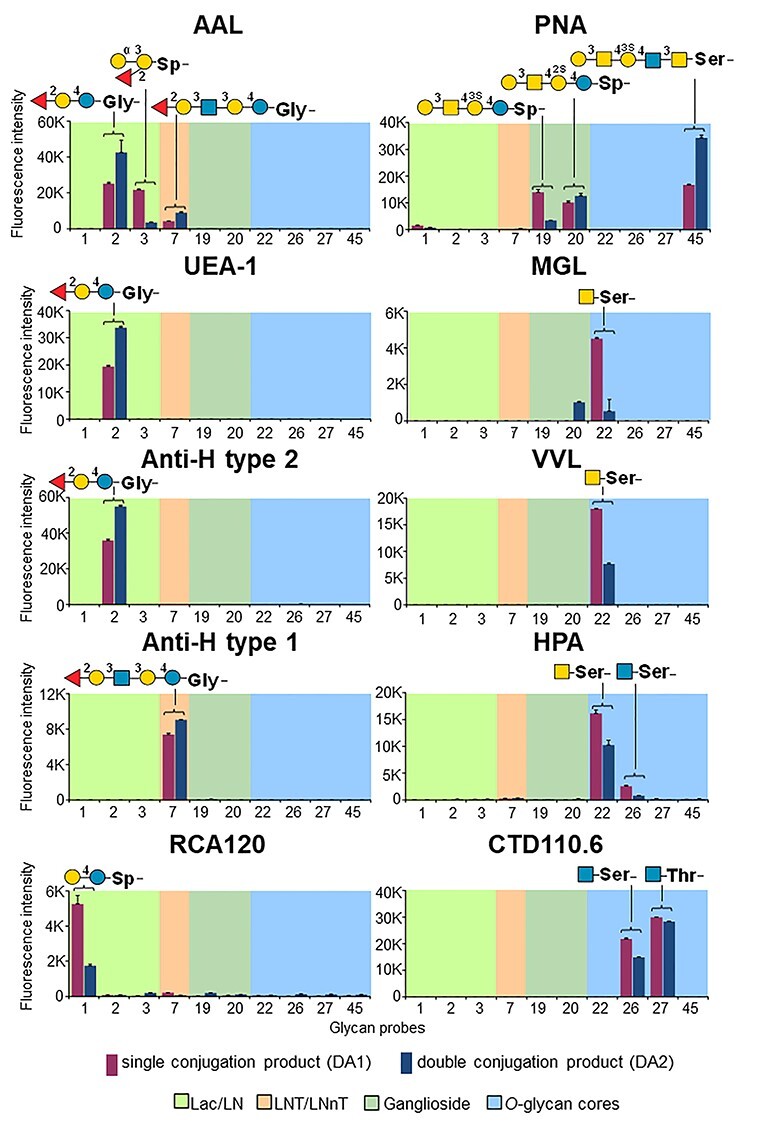
Comparison of the binding signal intensities of NGL products with single (DA1) or double (DA2) lipid chains immobilized noncovalently on nitrocellulose-coated slides. The probes are arranged according to their backbone-sequence type: lactose and *N*-acetyl lactosamine (Lac/LN), lacto-*N*-tetraose and lacto-*N*-neo-tetraose (LNT/LNnT), glycolipid and *O*-glycan core. The glycan sequence of probes eliciting binding signals are annotated (a more comprehensive comparison is shown as a heatmap, Figure S4). The representation of glycans follows the guidelines of Symbol Nomenclature for Glycans (Varki et al. 2015). The binding signals are means of fluorescence intensities of duplicate spots at 5 fmol of probe arrayed (with error bars) and are representative of at least two independent experiments. Binding signals are in red for the DA1 and blue for the DA2 conjugation products, respectively. The chart position assigned to each probe is referenced in Table SI (NGL Chart Pos.).

As methylamino-terminating glycan analogs are not readily available, we evaluated the binding signals elicited by NGLs with single or double lipid chains. The isolated products were arrayed and the binding with 10 carbohydrate sequence-specific proteins were analyzed (Table SII). The binding patterns with the DA1- and DA2-NGL pairs immobilized noncovalently on nitrocellulose-coated slides were similar overall (Figure S4, selected shown in Figure 3) and were consistent with prior knowledge of glycan recognition by these proteins (Table SII). These included binding by the α-fucose-specific proteins, *Aleuria aurantia* lectin (AAL), *Ulex europeus* agglutinin (UEA-1) and the anti-blood group H type 1 and H type 2 antibodies; the β-galactoside specific *Ricinus communis* agglutinin I (RCA_120_); the core 1 specific peanut agglutinin (PNA); the α-GalNAc-specific proteins, human macrophage galactose-type lectin (MGL), *Vicia villosa* lectin (VVL) and *Helix pomatia* agglutinin (HPA); and the *O*-β-GlcNAc-specific antibody CTD110.6 (Figure 3).

### Construction and validation of DA-NGL microarray using sequence-specific carbohydrate-binding proteins

Although as shown above the two or three different forms of DHPA-NGLs gave similar binding activities and these can be combined and used as the probes for microarray construction, NGLs of the DA1 series were used to construct the initial microarray for further evaluation. This contained 60 structurally diverse glycan sequences comprising both mucin-type *O*-glycan cores and *O*-GlcNAc linked to Ser/Thr, blood group-, *N*-glycan-, glycosaminoglycan- and glycolipid-related sequences, and β1,3-gluco-oligosaccharides (linear or branched) with degree-of-polymerization (DP) of 12, 13 and 15 (position #1-#60 Table SI), referred to as DA-NGL microarray hereinafter.

**Fig. 4:**
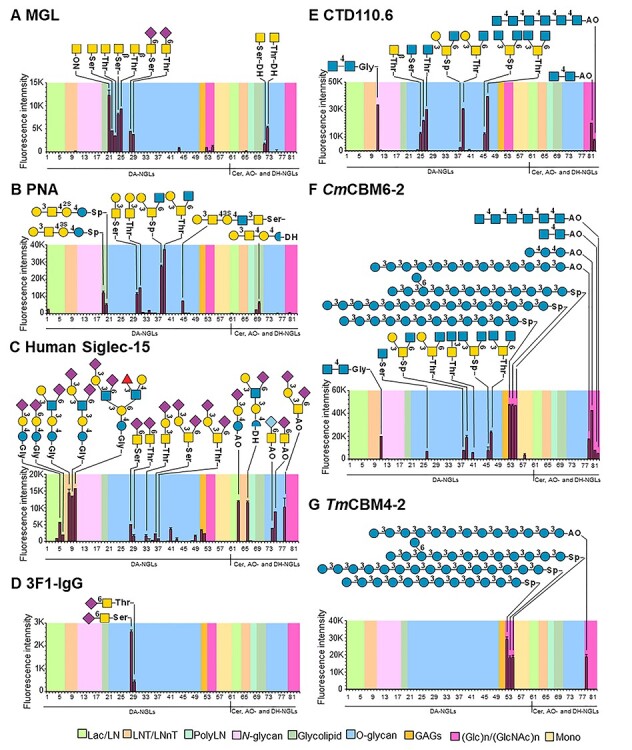
Glycan DA-NGL microarray validation using sequence-specific proteins. The microarray was probed with (**A**–**C**) lectins, (**D** and **E**) monoclonal antibodies and (**F** and **G**) carbohydrate-binding modules (CBMs) of bacterial glycoside hydrolases (see also Figure S5 for additional analysis). The probes are arranged according to their backbone-sequence type as indicated in the colored panels: lactose and *N*-acetyl lactosamine (Lac/LN), lacto-*N*-tetraose and lacto-*N*-neo-tetraose (LNT/LNnT), poly *N*-acetyllactosamine (PolyLN), *N*-glycans, glycolipid, *O*-glycan core, glycosaminoglycans (GAGs), glucose and *N*-acetylglucosamine homo-oligomers (Glc_n_/GlcNAc_n_), and monosaccharides (Mono). The representation of glycans follows the guidelines of Symbol Nomenclature for Glycans (Varki et al. 2015). The binding signals are means of fluorescence intensities of duplicate spots at 5 fmol of probe arrayed (with error bars) and are representative of at least two independent experiments. The chart position assigned to each probe is referenced in Table SI (NGL Chart Pos).

The DA-NGL microarray was probed with lectins, antibodies and carbohydrate-binding modules (CBMs) with known specificities (Table SII). The microarray analyses showed a good correlation of the binding profiles to the DA-NGLs with the reported carbohydrate-binding for the proteins analyzed (Figures 4 and S5), and these were similar to those obtained to the conventional NGLs or glycolipids (position #61-#82), included as reference probes. These results validated the DA-NGLs for binding studies. In addition, DA-NGL microarrays provided new information on the fine specificities of the proteins.

#### Mucin-type *O*-glycan core sequences and O-GlcNAc

The GalNAcα-Ser/Thr (Tn antigen) specific lectins human MGL (Figure 4A), VVL/VVA and HPA (Figure S5A-B) showed binding to all the GalNAcα1-terminating probes (#21-#23, Table SI). MGL also bound to GalNAcβ1-Ser/Thr (#24 and #25) and to the GalNAcα1-Ser/Thr substituted with an α2,6-linked Neu5Ac (α2,6-sialyl-Tn antigen) (#28, #29), in accord with published data (Mortezai et al. 2013). The core 1 specific lectin PNA (Figure 4B) showed binding to Galβ1–3GalNAcα1-Ser/Thr (#30, #31) and to the probes containing this epitope at the nonreducing terminal (#19, #20 and #45). Noteworthy, PNA showed a strong binding to core 2 Galβ1–3(GlcNAcβ1–6)GalNAcα1- sequence linked to either an aminopropyl linker (#38) or to Thr (#39).

The α2,6-sialyl-Tn antigen has been identified as a ligand for human Siglec-15 (Angata et al. 2007; MacAuley et al. 2014). Here, human Siglec-15 was demonstrated to bind to sialylated glycans with other backbone-types (Figure 4C). The α2,3- and α2,6-sialyl lactose (#5, #6) were bound with similar intensities as α2,6-sialyl-Tn (#28, #29) and related O-glycan probes (#33 #36, #37), whereas stronger binding was observed to the extended α2,6-sialylated LSTb, α2,3/α2,6-disialylated DSLNT and DSMFLNH probes (#8-#10). In contrast, the anti-sialyl-Tn specific mAb 3F1-IgG (Figure 4D) showed restricted binding to the two α2,6-sialyl-Tn probes (#28, #29) with a clear preference for α2,6-sialyl-Tn-Ser.

The *O*-GlcNAc mAb CTD110.6, showed strong binding to GlcNAcβ-Ser/Thr (#26, #27, Figure 4E) and exhibited cross-reactivity with chitobiose (GlcNAcβ1-4GlcNAc, #11) and GalNAcβ-Thr (#25), as reported previously (Reeves et al. 2014). The antibody also showed strong binding to core 2 (#39) and to GlcNAcβ6/β3-terminating core 4 (#46, #47) with a preference for Thr over the aminopropyl linker.

#### Peripheral Fuc-, Gal-, Man- or GlcNAc-sequences with different backbones

The terminal Manα1-, Galβ1- and GlcNAcβ1,4-probes also elicited specific binding signals with the plant lectins Concanavalin A (ConA), RCA_120_, and wheat germ agglutinin (WGA), respectively (Figure S5C–F). Additionally, ConA showed binding to the O-mannosylated peptide (#50) and WGA to probes with mucin *O*-glycan core sequences, albeit weakly: GlcNAcβ1–6-terminating core 2 (#38, #39), GlcNAc1-β6/β3- terminating core 4 (#47), and α2,6-sialyl-Tn-Ser/Thr (#28, #29). The fucose-specific lectin AAL and the anti-blood group H type 1 and type 2 antibodies showed the specific binding to the terminal fucosylated DA-NGL probes (Figure S5F–H). AAL also showed binding to the core-fucosylated *N*-glycan probes with fucose α1,3- and/or α1,6-linked to GlcNAc (#12-#14 and #18).

#### β1,3-Glucan sequences

The two bacterial CBMs showed different binding profiles in the DA-NGL microarrays, in accord with their specificities toward β1,3-glucans (Palma et al. 2015) and topologies of the binding sites (Table SII). *Cm*CBM6–2 bound with similar intensities to the linear β1,3-glucan probes with DP12 and DP15 (#53 and #54) and to the branched β1,3/1,6-glucan DP13 (#55) (Figure 4F), whereas *Tm*CBM4–2 showed stronger binding to the probe with the linear DP 12 (Figure 4G), compared to the branched probe with the same C5 linker. A new finding for *Cm*CBM6–2 was the consistent binding pattern observed to all GlcNAcβ1-terminating probes: chitobiose (#11), GlcNAcβ1-*O*-Ser (#26), and GlcNAcβ1-terminating core 2 (#38, #39), core 3 (#41) and core 4 (#46, #47).

### Application of DA-NGL microarray to derive specificities of glycan–protein interactions

As the glycan probe repertoire in the NGL microarray system has now been increased to include synthetic amino-terminating glycans, it allowed a more comprehensive study and better understanding of glycan–protein interactions than in the previous investigations using conventional NGL arrays as exemplified by the mammalian immune receptor Dectin-1 (Brown and Gordon 2005; Palma et al. 2006) and the VP8* domain of rotavirus [P19] (Liu et al. 2016; Li et al. 2018).

#### Dectin-1 binding to β-glucans

**Fig. 5:**
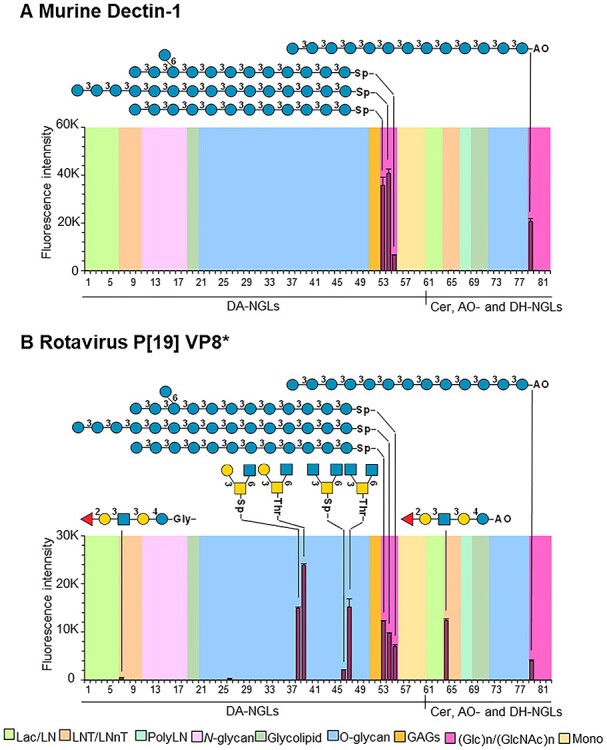
Application of DA-NGL microarray to derive specificities of glycan–protein interactions. (**A**) Murine Dectin-1; (**B**) Rotavirus [P19] VP8*. The probes are arranged according to their backbone-sequence type as in Figure 4. The glycan sequence of probes eliciting binding signals is annotated. The representation of glycans follows the guidelines of Symbol Nomenclature for Glycans (Varki et al. 2015). The binding signals are means of fluorescence intensities of duplicate spots at 5 fmol of probe arrayed (with error bars) and are representative of at least two independent experiments. The chart position assigned to each probe is referenced in Table SI (NGL Chart Pos).

Dectin-1 interaction with glucan polysaccharides is highly specific for backbone sequences of β1,3-linked glucose with a minimum chain length of DP10 (Palma et al. 2006, 2015). There has been evidence suggestive of the additional involvement of β1,6-glucosyl branching in Dectin-1 specificity (Adams et al. 2008), but there have been no direct binding data yet to support this, mainly because of the unavailability of sequence-defined long chain β1,3/1,6-branched gluco-oligosaccharides. Isolation and purification of branched long chain gluco-oligosaccharides from β-glucan polysaccharides has been difficult (Palma et al. 2006, 2015). Here, chemically synthesized, amino-terminating linear β1,3-gluco-oligosaccharides with DP12 and DP15 (#53 and #54, respectively) and a branched DP13 (#55) were used after their conversion into DA-NGLs and probed for Dectin-1 binding (Figure 5A). The results clearly showed that at these oligosaccharide chain lengths the strongest binding of Dectin-1 was to the linear β1,3-gluco-oligosaccharides, whereas the binding to the branched probe was markedly reduced. This unequivocally shows the chain length dependency and a negative influence of a β1,6-monoglucosyl branch closer to the nonreducing end on Dectin-1 binding.

#### Rotavirus P[19] VP8* binding to mucin O-glycan cores and blood group H type 1

The rotavirus P[19] VP8* specificity toward glycans has been investigated earlier in independent studies and it has been shown toward both mucin *O*-glycan cores and the blood group H type 1 sequence (Liu et al. 2016, 2017b; Sun et al. 2018). Although conventional NGL microarray screening analysis has given useful information on the specificity of this VP8*, it was not possible to directly compare the binding of the P[19] VP8* to these different types of glycans as the Ser/Thr-terminating mucin cores could not be prepared as NGL probes. In the present DA-NGL microarrays (Figure 5B) containing both types of glycan probes, the P[19] VP8* bound predominantly to the mucin core 2 (#38, #39) and core 4 (#46, #47), which share the core structure (GlcNAcβ1–6GalNAcα-), but not to core 1 or core 3 providing evidence for the role of the β1,3-linked Gal and β1,6-GlcNAc for the interaction (Liu et al. 2016). In comparison, the LNFP-I pentasaccharide probe (#7), which presents the blood group H type 1 sequence Fucα1-2Galβ1–3GlcNAcβ1–3Gal- bound by P[19] VP8* (Liu et al. 2016), elicited only a weak binding signal. The earlier observation of unpredicted binding of P[19] VP8* to β1,3-glucan sequences using conventional NGLs (Li et al. 2018) is also supported by the strong binding to probes #53-#55 in the DA-NGL microarray (Figure 5B).

### Comparison of binding signals with the noncovalent NGL and covalent microarrays

**Fig. 6:**
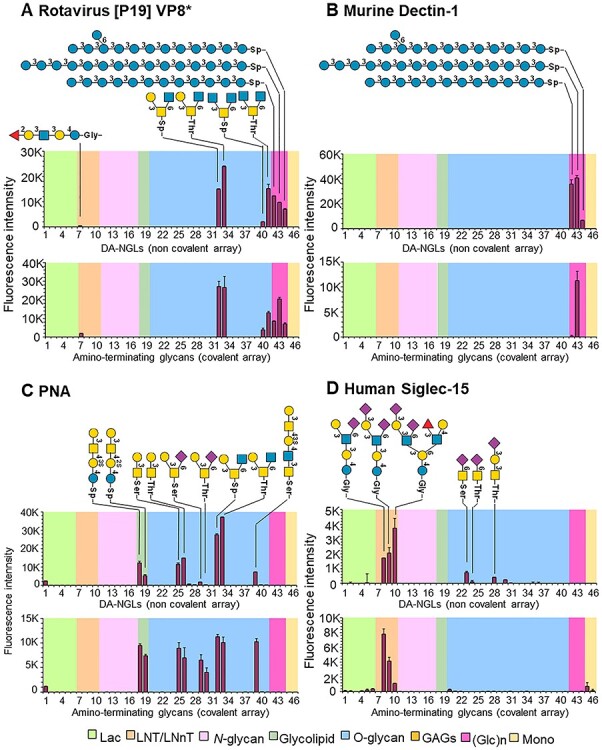
Comparison of noncovalent NGL and covalent microarrays. The comparison is illustrated by four typical examples to highlight the binding by (**A**) rotavirus [P19]VP8*; (**B**) murine Dectin-1; (**C**) PNA; and (**D**) human Siglec-15 (see also Figure S6A-N for a more comprehensive comparison). The probes are arranged according to their backbone-sequence type as in Figure 4 and 5. The glycan sequence of probes eliciting binding signals is annotated. The representation of glycans follows the guidelines of Symbol Nomenclature for Glycans (Varki et al. 2015). The chart position assigned to each probe is referenced in Table SI (Covalent/NGL Chart Pos).

A panel of 46 amino-terminating sugars used to prepare DA-NGLs, including the neutral, fucosylated and sialylated glycans, high-mannose and bi-antennary *N*-glycans, glucan oligosaccharides, and Ser/Thr-terminating mucin *O*-glycan cores, were selected for covalent printing onto NHS slides. These were assessed for comparison of the binding signals using the glycan-binding proteins analyzed in the DA-NGL microarrays (Table SI, covalent/NGL chart positions #1-#46). As shown in Figures 6 and S6A–N, similar binding profiles were observed using the two types of microarrays. Among the 18 proteins analyzed, three showed identical binding profiles: P[19]VP8* (Figure 6A) anti-STn 3F1 (Figure S6D) and anti-H type 2 (Figure S6I), 12 gave similar binding patterns with some minor differences in terms of the probes bound or the binding intensity: mDectin-1 (Figure 6B), PNA, (Figure 6C), MGL (Figure S6A), VVL/VVA (Figure S6B), HPA (Figure S6C), mAb CTD110.6 (Figure S6D), UEA-1 (Figure S6H), anti-H type 1 (Figure S6J), RCA-120 (Figure S6K), ConA (Figure S6L), *Cm*CBM6–2 (Figure S6M) and *Tm*CBM4–2 (Figure S6N). There were three that showed some major differences with respect to the glycan probes bound and signal intensity human Siglec-15 (Figure 6D), WGA (Figure S6F) and AAL (Figure S6G).

These findings are highlighted in the four selected typical examples shown in Figure 6. In all four examples, the binding specificities and binding signals detected are the same. Rotavirus [P19] VP8* showed similar binding in both platforms in terms of both binding patterns and intensity values (Figure 6A). However, in some cases NGLs showed more intense signals, e.g. Dectin-1 binding to linear β1,3-gluco-oligosaccharides with DP12 and DP13 (#42 and #43, Figure 6B) and branched DP13 (#44), whereas in other cases covalent arrays exhibited binding to weaker binders that was only marginally detected in the DA-NGL arrays, e.g. PNA binding to 2,6-sialylated core 1 (#29, #30, Figure 6C). Human Siglec-15 is among the very few examples analyzed showing a difference between the two platforms: in the covalent array, Siglec-15 showed a restricted binding profile to the sialylated milk sugars LSTb and DSLNT (#8, #9, Figure 6D) with weak binding to the branched DSMFLNH probe (#10), whereas in the NGL arrays exhibited similar binding to these three probes, in addition to the binding detected to α2,6-sialyl-Tn-Ser (#23) and α2,3-sialyl core 1-Thr (#28) (in this comparison Siglec-15 was tested as a non-pre-complex and in Figure 4C as a pre-complex with the detection antibody, which enhances the binding signal).

It is also interesting to note that in most cases NGL array showed 3–6 times more intense binding signals than the covalent arrays while the background of covalent arrays is generally lower than that of NGL array, although there are seven cases in which comparable binding intensities were observed in the two platforms.

## Discussion

With the new lipid reagent, we have demonstrated that the widely used synthetic amino-terminating glycans are well suited to the NGL-based microarrays and that the NGL probe library of one of the major international microarray resources can be much expanded in repertoire. The glycan structures analyzed in this initial proof-of-concept study, although limited in number, cover different glycan structural types, e.g. mucin-type *O*-glycan cores and *O*-GlcNAc linked to Ser or Thr, blood group antigens and ganglioside-, *N*-glycan- and glucan-related sequences. The utility of the DA-NGL microarray was demonstrated by the specific binding patterns obtained with plant and mammalian lectins, monoclonal antibodies and CBMs.

With the probe types extended to the short chain *O*-glycans with intact core GalNAc and the linked Ser/Thr residues, we were able to broaden the knowledge on the specificities of glycan-binding proteins previously not available. We showed that PNA lectin, widely used in the detection of core 1 T-antigen, exhibits strong binding to the core 2 antigen and that the *O*-GlcNAc-specific antibody CTD110.6 and *C. mixtus Cm*CBM6–2 can accommodate core 2 and core 4 antigens, through recognition of the terminal GlcNAcβ1- residues. In addition, here and in a recent published study (Murugesan et al. 2021), we demonstrated that human Siglec-15 can interact with α2,3 and α2,6 sialylated glycan structures other than the tumor-associated α2,6-sialyl-Tn, showing strong binding to structures with Neu5Ac α2,6-linked to an internal GlcNAc. Human Siglec-15 has recently gained research interest as its function is important for promoting a tumor immunosuppressive phenotype and tumor progression (Wang et al. 2019) and for osteoclast biology (MacAuley et al. 2014). Our results open the way to studies of the implication of recognition of sialyl glycans other than α2,6-sialyl-Tn antigen in the function of this Siglec.

The specific binding observed with the 3F1 mAb against the α2,6-sialyl-Tn *O*-glycan corroborates recent studies on the L2A5 antibody that is being developed for anti-cancer immunotherapy (Loureiro et al. 2018). Noteworthy, in our analysis of 3F1 and L2A5 antibodies, we observed a clear preference for the α2,6-sialyl-Tn glycan in Ser over Thr. The preference for Ser or Thr *O*-glycans has been reported for proteins targeting the Tn *O*-glycan antigen: while some anti-Tn antibodies (Coelho et al. 2015) and HPA lectin (Madariaga et al. 2014) prefer Tn-Ser, others such as anti-MUC1 antibodies (Martínez-Sáez et al. 2015) and VVL (Madariaga et al. 2014) have a higher affinity for Tn-Thr. Indeed, our microarray data also show a preference of VVL for Tn-Thr. Published structural data showed that the Tn-Ser and Tn-Thr structures adopt different conformations in solution and in the protein-bound state, allowing to establish specific glycan and water-mediated interactions with the protein-binding site (Bermejo et al. 2018). Our results further support the hypothesis that the preference of binding to the Ser or Thr structures may add to the specificity of a given glycan-binding protein and have biological significance in the molecular recognition of natural *O*-glycans.

Dectin-1 is the major receptor for β-glucans on macrophages. The recognition of β-glucans promotes oligomerization of the receptor at the cell surface and mediates cell signaling in the immune cell response to several fungal species (Plato et al. 2013). The interaction of Dectin-1 with β-glucans and the consequent cellular effects are thought to be dependent on the linkage, size and branching (Adams et al. 2008; Marakalala et al. 2011). Although the specificity for the β1,3-linkage and the chain length requirement for Dectin-1 binding have been well accepted using glucan-derived oligosaccharides, the influence of β1,6-branching of the glucan chain has needed corroboration (Adams et al. 2008; Palma et al. 2015). The microarray analysis reported here have enabled direct comparison of glucan-derived linear and chemically synthesized β1,6-branched gluco-oligosaccharides with identical backbone; our results show that a β1,6-linked glucose positioned at the nonreducing penultimate glucose has a damping effect on Dectin-1 binding of. The monoglucosyl branching at this position likely interferes with the presentation of the hypothesized helical conformational epitope formed by the β1,3-linked glucose chain recognized by Dectin-1. It will be important to investigate the influence of other β1,6-branches on short and long β1,3-linked glucose backbone chains. This will lead to a better understanding of the molecular basis of the recognition of fungi by Dectin-1.

Rotaviruses comprise a genotypically variable family of viruses that cause severe gastroenteritis in human and animals and use glycans as receptors for infection. The recognition of glycans by rotaviruses in a genotype-dependent manner is via the distal VP8* head of the spike protein VP4. For P[19] genotype, the glycan specificity of VP8* has been assigned to mucin *O*-glycan cores (particularly core 2) and H type 1 histo-blood group antigen (HBGA) precursors using different types of microarrays (Liu et al. 2016; Li et al. 2018). Here, we were able to compare directly the binding of P[19] VP8* to these types of antigens prepared as probes using the same lipid reagent for presentation on the microarray. The predominant binding was observed to mucin cores 2 and 4, with only weak binding to LNFP I presenting the H type 1 HBGA trisaccharide epitope (Fucα1-2Galβ1–3GlcNAcβ1-) required for binding. The results are in accord with solution NMR data (Liu et al. 2016) and also evidenced from earlier glycan microarray data (Liu et al. 2016; Sun et al. 2018). This evidence poses important questions on the functional significance of the preferential binding to the mucin core *O*-glycans for viral pathogenesis compared with H HBGAs. P[19] rotavirus commonly infect animals (porcine) and only sporadically humans. It is postulated that the P[19] genotype may represent an early evolutionary stage that started adapting to human receptors but retaining the binding specificities to the short chain mucin cores 2 and 4, and also H type 1 HBGAs. A more recent study using microarrays of *O*-glycans isolated from a porcine mucin by the beam search strategy (Li et al. 2018) showed a more potent P[19] VP8* binding to the extended H type 1 chain (Fucα1-2Galβ1–3GlcNAcβ1–3Galβ1-4GlcNAcβ1-?Gal-). The reported crystal structures of the P[19] VP8* in complex with LNFP-I and core 2-Thr showed that both ligands are bound in a similar way but that the binding site is able to accommodate an extended glycan chain (Liu et al. 2017b). Thus, further studies with structurally diverse elongated mucin *O*-glycans with intact cores and HBGA sequences are required to clearly answer VP8* specificity for P[19] infection and rotavirus evolution. The observation that P[19] VP8* also exhibits binding to non-mammalian β1,3-glucan sequences is consistent with previous microarray data (Li et al. 2018). These sequences are typical and highly abundant in fungal cell walls and act as pathogen-associated molecular recognition patterns (PAMPs) (Brown and Gordon 2005). The glycan microarray data may be the first indication of interactions of enteric viruses with fungi within the intestinal microbiome, which deserves further investigation.

The ability to prepare NGLs from the amino-terminating glycans made it possible for the very same glycan molecules to be used in the two major platforms (Scheme 1). This is important for the comparison of the two platforms as this eliminates variables that may influence the binding results (Wang et al. 2014), e.g., the origin, the quality and quantity of glycan molecules used in different arrays. The data presented here showed, for the first time, that two of the major glycan microarray platforms and employed by the international resources give similar binding profiles with the different glycan-binding proteins analyzed.

## Conclusions

Covalent and non-covalent glycan immobilization are the two different principles to construct glycan microarrays represented by the major international microarray resources. Here, using the NGL-based noncovalent microarray as the example, we demonstrate the benefit of combined chemical strategies taking both advantages of glycans of aldehyde-terminating from natural glycomes and amino-terminating by synthetic means to increase size and diversity of glycan probe libraries. We were able to use the very same glycans in both covalent and noncovalent platforms (Scheme 1) enabling a comparison of their performance. The analyses in the two types of microarrays demonstrate similar binding profiles with different classes of glycan-binding proteins and provide knowledge on the unique specificity of the immune receptor Dectin-1 toward β-glucans and core *O*-glycan recognition by the adhesive protein VP8* of the rotavirus P[19]. The method can now be applied to expand the glycan library coverage in noncovalent microarrays by incorporating probes conventionally listed in the covalent arrays prepared by chemical or chemoenzymatic synthesis, including glycopeptides. This will broaden application of glycan microarrays and provide better opportunities to decipher glycan recognition systems with implications in understanding cellular mechanisms in health and disease.

## Materials and methods

### Materials

DHPE was from Fluka (Dorset, UK). Sodium cyanoborohydride (NaBH_3_CN) was from Acros Organics (New Jersey, USA). 1-Ethyl-3-(3-dimethyl aminopropyl) carbodiimide hydrochloride (EDC), *N*-hydroxybenzotriazole (HOBT) and *N*, *N*-diisopropylethylamine (DIPEA) were from Aladdin (Shanghai, China). All other chemicals were analytical reagent grade. HPTLC plates were from Merck (Darmstadt, Germany). Column chromatography was conducted by elution of a column of silica gel (200–300 mesh). ^1^H and ^13^C NMR spectra were recorded with a JEOL JNM-ECP-600 (600/150 MHz) or an Agilent DD2–500 (500/125 MHz) instrument. Chemical shifts were reported on the *δ* scale. CDCl_3_ (*δ* = 7.26 ppm) or tetramethylsilane (*δ* = 0.00 ppm) was used as an internal reference. ESI-MS of synthetic amino-terminating saccharides were carried out on a Waters Q-TOF-type mass spectrometer (Manchester, UK).

### Amino-terminating glycans

The structures together with the sources of the 60 amino-terminating glycans used to prepare DA-NGLs and for covalent microarrays are given in Table SI. The chemical synthesis of Gal–C2–NH_2_, Gal–Ph–NH_2_, Glc–C2–NH_2_, Man–C2–NH_2_, Xyl–C2–NH_2_, Lac–C2–NH_2_, SM1a–C3–NH_2_ and SM1a(2S)–C3–NH_2_, and methylamino-terminating sugars Glc–C3–NHMe, Gal–C3–NHMe and Lac–C2–NHMe is described in Supplementary Methods. The glycine-terminating glycans as *N*-glycosides were prepared by conjugation of the respective reducing sugars with glycine as described (Likhosherstov et al. 2015). Mono- and di-sialyl Core 1 linked with either a serine and threonine were isolated from human urine and purified by HPLC as described (Parkkinen and Finne 1983). GalNAcα-Ser, GalNAcα-Thr, GalNAcβ-Ser, GalNAcβ-Thr, GlcNAcβ-Ser, GlcNAcβ-Thr, were synthesized as described in Supplementary Methods. The linear and branched gluco-oligosaccharides Glc12-C5-NH_2_ and Glc13(B10)-C15-NH_2_ were synthesized using solid-phase chemistry by automated carbohydrate synthesizer as described (Weishaupt et al. 2013; Weishaupt et al. 2017).

Glc15-C2-NH_2_ was chemically synthesized and provided by Novartis Pharmaceuticals as a gift. Heparin tetrasaccharides Hep-4-NAc-Ph-NH_2_ and Hep-4-NS-Ph-NH_2_ were synthesized chemoenzymatically and provided by Jian Liu. Core 2-Sp and Core 4-Sp and were provided by Dr Nicolai Bovin. 3’SA-Lac-C2-NH_2_, B-Tri-C3-NH_2_ and GalNAc-ONH_2_ were purchased from Elicityl (Crolles, France), and Core 1-Ser was from Dextra (Reading, UK). Fully protected, Core 1-Thr and Core 4-Thr were from Sussex Research (Ottawa, Canada) and used after deprotection.

### Synthesis of carboxyl-functionalized phospholipid reagent *N*-(4-oxobutanoic acid)- 1,2-dihexadecyl-*sn*-glycero-3-phosphoethanolamine (DHPC)

DHPE (20 mg, 30 μmol) was dissolved in 8 mL tetrahydrofuran, followed by addition of succinic anhydride (4 mg, 45 μmol) and triethylamine (5 μL, 45 μmol). The reaction mixture was stirred for 2 hr at 50°C. The completion of the reaction was indicated by TLC analysis (CHCl_3_/MeOH, 6:1). The mixture was then concentrated before dilution with hydrochloric acid (15 mL). The reaction product was extracted with CHCl_3_ (10 mL× 3). The combined organic extracts were dried with Na_2_SO_4_, and concentrated to afford compound DHPC (22.4 mg, 97%) as a white solid. ESI-MS: calculated for C_41_H_81_NO_9_P [M-H]^−^ 762.5649, found *m*/*z* 762.5682.

### Synthesis of aldehyde-functionalized phospholipid reagent *N*-(4-formylbenzamide)- 1,2-dihexadecyl-*sn*-glycero-3-phosphoethanolamine (DHPA)

4-Carboxybenzaldehyde (12 mg, 80 μmol) was dissolved in 10 mL CH_2_Cl_2_. DIPEA (15 μL, 90 μmol), EDC (Sigma, 17.5 mg, 90 μmol), HOBT (Sigma, 12.2 mg, 90 μmol) and DHPE (20 mg, 30 μmol) were sequentially added at 0°C. The reaction mixture was stirred for 30 min, followed by 24 h at room temperature. HPTLC of an aliquot of the reaction mixture revealed a major product, R*_f_* 0.3 (developed with CHCl_3_/MeOH, 6:1), visualized under 254 nm UV light or under 365 nm UV light after primulin staining. The mixture was concentrated, and the residue was purified by silica gel chromatography (CH_2_Cl_2_/MeOH, 20:1–15:1) to give DHPA (15 mg, 63%) as a white solid. ^1^H NMR (500 MHz, CD_3_OD/CDCl_3_ = 1:2): δ 10.03 (s, 1H, –CHO), 8.02–7.99 (m, 2H, Ph–H), 7.94–7.91 (m, 2H, Ph–H), 7.48 (s, 1H, CONH–), 4.04–3.98 (m, 2H), 3.89–3.83 (m, 2H), 3.65–3.60 (m, 2H), 3.56–3.46 (m, 4H), 3.43–3.36 (m, 3H), 1.53–1.46 (m, 4H), 1.30–1.16 (m, 52H), 0.84 (*t*, *J* = 6.9 Hz, 6H, –CH3); 13C NMR (125 MHz, CD3OD/CDCl3 = 1:2): δ 192.19, 167.47, 138.91, 138.16, 71.69, 70.57, 70.08, 64.97, 63.79, 40.84, 31.79, 29.81, 29.56, 29.52, 29.51, 29.47, 29.39, 29.22, 25.95, 25.89, 22.52 and 13.73. HR ESI-MS: calcd. For C45H81NO8P [M-H]^−^ 794.5700, found *m*/*z* 794.5765.

### Preparation of DHPA (DA)-NGLs

Typically, amino-terminating saccharides (20–50 nmol) were incubated with 10 equivalents of DHPA and 15 equivalents of reducing reagent NaBH_3_CN (except for GalNAc-ON). For example, 200 nmol DHPA (50 μL of 4 mM in CHCl_3_/MeOH, 1:1, or in CHCl_3_/EtOH, 1:1) was added to 20 nmol dried saccharide in a glass microvial. The mixture was evaporated to dryness under a nitrogen stream and dissolved in 50–100 μL CHCl_3_/MeOH or CHCl_3_/EtOH (1:1). The mixture was incubated at 60°C for 6–24 h. For the aromatic amine-functionalized glycans (Hep-4-NS-PhNH_2_ and Hep-4-NAc-PhNH_2_), incubation was conducted at 80°C for 48 hr. Aliquots of the reaction mixtures were analyzed by HPTLC using aluminum-backed silica gel plate (Merck) and solvent system CHCl_3_/MeOH/H_2_O (65:35:8) with primulin and orcinol staining (Chai et al. 2003). Aiming to minimize conjugation of two lipids per glycan, different reaction conditions were also attempted; these included lower ratio of reagent (e.g. sugar: reagent, 1:2 and 1:4), different conjugation temperatures (45°C and 80°C) and with inclusion of 5% v/v water.

DA-NGLs were isolated from reaction mixtures by semipreparative TLC or silica cartridge (Sep-Pak, Waters) as described (Chai et al. 2003). Purified DA-NGLs were analyzed by HPTLC and MALDI-MS, and quantified after primulin staining using Lac-DA as the standard. Lac-DA was previously quantified using the conventional Lac-DH NGL by orcinol staining (Chai et al. 2003). A factor of 1.5 was used for DA-NGLs with two lipid tails, which was obtained by primulin and orcinol quantitation of Lac-DA and Lac-DA2. DA-NGLs were stored at −20°C in CHCl_3_/MeOH/H_2_O (25:25:8) until analysis.

### Mass spectrometry

MALDI-MS of the DHPA-NGLs was carried out on an AXIMA Assurance linear TOF instrument (Shimadzu) and MALDI-CID-MS/MS on an AXIMA Resonance QIT-TOF instrument (Shimadzu). NGLs were dissolved in a solvent of CHCl_3_/MeOH/H_2_O (25:25:8) at ~10 pmol/μl and 0.5–1 μL was deposited on the sample target together with 1 μL of matrix of 2′,4′,6′-trihydroxyacetophenon. For MS the laser energy at an attenuation scale 80 and for CID-MS/MS a collision gas Ar (2 bar) and collision energy at 80–140, depending on specific samples, were used.

### Construction of microarrays

Information on the glycan probes, generation of the microarrays, imaging and data analysis are described in the Supplementary MIRAGE (Liu et al. 2017a) document (Table SIII). The different procedures for construction and analysis of the two microarrays are briefly described below.

For construction and validation of the noncovalent microarrays the 60 DHPA-NGLs derived from amino-terminating sugars with single lipid tail (Table SI) were used. As reference probes, 19 conventional NGL probes of the DH- or AO-types, and 3 glycosylceramides (Table SI) were used (Chai et al. 2003; Liu et al. 2007). The microarrays were prepared via noncovalent immobilization following established procedures (Liu et al. 2012). The NGL or glycolipid probes were printed at 2 and 5 fmol/spot in duplicate onto 16-pad nitrocellulose-coated glass slides (UniSart 3D Microarray Slide; Sartorius, Goettingen, Germany). The fluorescent dye Cyanine 3 was included in the printing solution as a tracer for quality control of arraying process and for localization of the printed spots.

For construction of the microarrays via covalent immobilization, 46 of the amino-terminating sugars (Table SI) were printed directly onto 16-pad NHS-activated glass slides (Schott Nexterion H; Jena, Germany) using protocols published previously (Blixt et al. 2004; Smith et al. 2010). In brief, the probes were printed at a concentration of 100 μM (330 pl and 33 fmol/spot) in the printing buffer (100 mM phosphate buffer, pH 8.7), in 4 replicates. The Alexa Fluor 647-labeled streptavidin (100 μM in 100 μg/ml BSA in printing buffer) was used as a grid alignment control. Printing was carried out at 60% relative humidity, followed by probe immobilization overnight on the arrayer slide deck at 80% relative humidity in the dark. The remaining reactive NHS groups were blocked by immersion in 50 mM ethanolamine in 100 mM borate buffer pH 8.7 and incubation for 2 h. The slides were rinsed 3 times with 100 mM Phosphate Buffer Saline pH 7.4 with 0.05% Tween 20 (PBST), followed by rinsing with water.

### Analysis of protein binding in microarrays

The protein samples analyzed, their preparation, concentration and detection antibodies are detailed in Table SII. The microarray binding assays were performed, following established protocols for NGL-based microarrays (Liu et al. 2012, 2018) or CFG-type covalent microarrays (Blixt et al. 2004; Smith et al. 2010), with minor differences described below.

The plant lectins were analyzed using a single-step overlay protocol for biotin-tagged samples. In brief, the DA-NGL subarrays were blocked with 3% bovine serum albumin (BSA, A8577 Sigma) in 20 mM Tris–HCl pH 8.5, 150 mM NaCl, 2 mM CaCl_2_ and 2 mM MgCl_2_ (3% BSA/TBS-Ca-Mg), followed by incubation for 2 h with the different lectin solutions, prepared in the binding buffer (1% w/v BSA in TBS-Ca-Mg).

The antibodies were analyzed using specific biotinylated secondary antibodies for detection. In brief, after blocking with 3% BSA in 100 mM phosphate-bufferred saline pH 7.4 (3%BSA in PBS), the microarrays were probed for 2 h with the solutions of the antibodies prepared in binding buffer (1% BSA in PBS), followed by incubation for 1 h with 10 μg/ml biotinylated anti-mouse IgG in the binding buffer.

The His-tagged CBMs were analyzed at a final concentration of 10 μg/mL pre-complexed with mouse monoclonal anti-poly-histidine (Ab1) and biotinylated anti-mouse IgG (Ab2) antibodies, both from Sigma, at a ratio of 1:3:3 (by weight). While blocking with 3% BSA in TBS-Ca-Mg, the CBM-antibody complexes were prepared by preincubating Ab1 with Ab2 for 15 min at ambient temperature, followed by addition of CBMs, incubation for a further 15 min, and diluted in the binding buffer (1% BSA in TBS-Ca-Mg) for microarray overlay. The binding of His-tagged murine Dectin-1 was detected following a similar procedure but without pre-complexing the protein with the detection antibodies: after overlaying with the Dectin-1 solution (30 μg/mL in 1% BSA in TBS-Ca-Mg) for 2 h, the slides were incubated for 1 h with the antibody pre-complexes at a final concentration of 10 μg/mL in the binding buffer.

The human Fc-tagged MGL was analyzed as a pre-complex with the biotinylated anti-human IgG (Vector) detection antibody (1:1 ratio, by weight). While blocking the slides with 3% BSA in TBS-Ca-Mg, the MGL-antibody complex was prepared by preincubating the protein and antibody for 1 h at 4°C and diluted at a final concentration of 10 μg/mL in the binding buffer (1% BSA in TBS-Ca-Mg) for microarray overlay. For analysis of Fc-tagged Siglec-15 two conditions were used: 1) the arrayed slides were blocked with 2%BSA in PBS and the protein was analyzed at 2 μg/mL as a pre-complex with the biotinylated anti-human IgG detection antibody (1:1 ratio, by weight), which was prepared by preincubating the protein and antibody, diluted in the final required volume of blocking buffer, for 1 h at 4°C; and 2) the arrayed slides were blocked with 3%BSA in PBS and the protein was analyzed at 20 μg/mL in the binding buffer (1%BSA in PBS) followed by detection with biotinylated anti-human IgG.

For analysis of the GST-tagged P[19] VP8* viral protein, the slides were blocked with 0.02% casein (Pierce) and 1% BSA in HBS (10 mM HEPES buffer pH 7.4, 150 mM NaCl) with 5 mM CaCl_2_. The protein was analyzed at 50 μg/ml in the blocking buffer, followed by detection with rabbit anti-GST polyclonal antibody (Santa Cruz) and biotinylated anti-rabbit IgG (Sigma), both at 1:200 in the blocking buffer.

The analysis of protein binding in covalent microarrays was carried out using the same procedures and buffer systems described above for the different proteins, but with the two differences: 1) omission of the 1 h blocking step and 2) addition of 0.05% Tween 20 to the binding and washing buffers. One exception was the GST-tagged P[19] VP8* protein that was analyzed in a binding buffer without casein (1% BSA in HBS with 2 mM CaCl_2_, 2 mM MgCl_2_, 0.05% Tween 20).

For all the analyses, the AlexaFluor-647-labeled streptavidin (Molecular Probes, 1 μg/ml) was used for fluorescence readout. Imaging and data analysis were essentially as described (Liu et al. 2012). The parameters for recording the fluorescence images were selected considering the signal to noise ratio, and saturation of the signal in the different experiments. These are detailed in the MIRAGE (Liu et al. 2017a) document (Table SIII). The binding signals in the DA-NGL microarray were dose-dependent. Results given are plotted as an average of two replicates for binding signals at 5 fmol per spot. The binding signals in the covalent microarray were plotted as the average of 4 replicates at 33 fmol/spot.

## Author contributions

W.C. conceived the project. C.L. designed the chemical strategies and A.S.P. designed and performed microarray binding experiments and carried out data analysis. P.Z. carried out the synthesis of the novel lipid reagents and related glycan standards. C.G., Z.L. and F.T. performed microarray binding experiments and contributed to data analysis. W.C. and Y.Z. carried out analysis of glycan probes and L.M.S. performed microarray printing. M.W., P.H.S., L.M.L., V.P., J.Y. and U.W. contributed to the key glycan probes. W.C., A.S.P. and C.L. wrote the paper and P.Z., J.Y. and U.W. wrote the synthetic part. All co-authors edited and approved the manuscript.

## Supplementary Material

DHPA-Supporting_Information-revised_cwab037

## Abbreviations

DA, aldehyde-functionalized phospholipid reagent *N*-(4-formylbenzamide)-1,2-dihexadecyl-*sn*-glycero-3-phosphoethanolamine; DHPE, 1,2-dihexadecyl-*sn*-glycero-3-phosphoethanolamine; DHPA, *N*-(4-formylbenzamide)-DHPE; DP, degree-of-polymerization; CBM, carbohydrate-binding module; Siglec, sialic acid binding Ig-like lectin; HBGA, histo-blood group antigen; AAL, *Aleuria aurantia* lectin; ConA, Concanavalin A; HPA, *Helix pomatia* agglutinin; MGL, macrophage galactose-type lectin; PNA, peanut agglutinin; RCA_120_, *Ricinus communis* agglutinin I; UEA-1, *Ulex europeus* agglutinin; VVL, *Vicia villosa* lectin; WGA, wheat germ agglutinin.
