# Supplementary material for: Noncovalent microarrays from synthetic amino-terminating glycans: Implications in expanding glycan microarray diversity and platform comparison
Source: Glycobiology. 2021 May 8;31(8):931–46. doi: 10.1093/glycob/cwab037 (PMC8434801; doi:10.1093/glycob/cwab037)
Supplement: DHPA-Supporting_Information-revised_cwab037 [file dhpa-supporting_information-revised_cwab037.docx]

**Supporting Information**

**Non-Covalent Microarrays from Synthetic Amino-Terminating Glycans -**

**Implications in Expanding Glycan Microarray Diversity and Platform Comparison**

Chunxia Li^1,2^*, Angelina S. Palma^3^*, Pengtao Zhang^1^, Yibing Zhang^4^, Chao Gao^4^, Lisete M. Silva^4^, Zhen Li^4^, Filipa Trovão^3^, Markus Weishaupt^5^, Peter H. Seeberger^5^, Leonid M. Likhosherstov^6^, Vladimir Piskarev^7^, Jin Yu^8^, Ulrika Westerlind^8^, Wengang Chai^4^**

^1^Key Laboratory of Marine Drugs, Ministry of Education, School of Medicine and Pharmacy and Shandong Provincial Key laboratory of Glycoscience and Glycoengineering, Ocean University of China, Qingdao, China; ^2^Laboratory for Marine Drugs and Bioproducts of Pilot National Laboratory for Marine Science and Technology (Qingdao), Qingdao 266237, China; ^3^Applied Molecular Biosciences Unit, Department of Chemistry, School of Science and Technology, NOVA University of Lisbon, Portugal; ^4^Glycosciences Laboratory, Imperial College London, London, United Kingdom; ^5^Department of Biomolecular Systems, Max-Planck-Institute of Colloids and Interfaces, Potsdam, Germany; ^6^N.D. Zelinsky Institute of Organic Chemistry, Russian Academy of Sciences, Moscow, Russia; ^7^Nesmeyanov Institute of Organoelement Compounds, Russian Academy of Sciences, Moscow, Russia;^8^Umeå University, Department of Chemistry, KBC-building, Linneaus väg 6, S-907 36 Umeå, Sweden.

*These authors contributed equally to this work.

**To whom correspondence should be addressed: Tel: +44-20 75942596; e-mail: [w.chai@imperial.ac.uk](mailto:w.chai@imperial.ac.uk)

Running title: Comparison of non-covalent and covalent glycan microarrays

**Content**

| **Figure S1** | Analysis of the DA-NGLs products |
| --- | --- |
| **Figure S2** | TLC analysis of conjugation products of Gal-C2-NH_2_ with DHPC and DHPA. |
| **Figure S3** | TLC analysis of DHPA conjugation product of GalNAcα1-O-NH2. |
| **Figure S4** | Comparison of the binding signals of NGL products with single (DA1) and double (DA2) lipid chains. |
| **Figure S5** | Quality control of DA-NGL probes with *O*-GalNAc core sequences and different backbone and peripheral Fuc-, Gal-, Man- or GlcNAc- sequences using plant lectins and antibodies. |
| **Figure S6** | Comparison of glycan-binding patterns in non-covalent and covalent microarray platforms. |
| **Table SI** | List of the probes in DA-NGL microarrays and in covalent microarrays. |
| **Table SII** | List of proteins investigated and their reported glycan recognition, sources and summarised information on the assay conditions and detection systems used for their analysis. |
| **Table SIII** | Supplementary glycan microarray document based on MIRAGE guidelines (doi:[10.3762/mirage.3](http://www.beilstein-institut.de/en/projects/mirage/guidelines#glycan_microarrays)). |
| **Supplementary Methods** |  |
| **Supplementary References** |  |

**Supplementary Figures**

**A**  DHPC conjugation

**B**

DHPA conjugation

**C** Proposed reaction mechanism of DHPC conjugation

excess

lipid reagent

(DHPA)_2_-NGL

origin

excess

lipid reagent

origin

DHPA-NGL

DHPC-NGL

DHPC-NGL

**primulin**

**orcinol**

**primulin**

**orcinol**

Reagents and condition used: a) Et_3_N, THF, 50^o^C, 97%; b) EDC, HOBt, DIPEA, CHCl_3_/CH_3_OH (1;1), r.t.

**Figure S1**. TLC analysis of conjugation products of Gal-C2-NH_2_ with DHPC and DHPA. With DHPC (A), multiple conjugation products were formed and low yield of NGL was obtained, as indicated by the large amount of unconjugated Gal-C2-NH_2_ at the bottom. The possible reason for the low yield is shown in (C). With DHPA (B), the yield of NGL product is higher (85%) with a ratio of single and double conjugation: 3:2.

**A**

**C**

**B**

**Figure S2**: Analysis of the DA-NGLs products. (A) TLC analysis of DHPA conjugation product of Gal-C2-NH2; (B) MALDI-MS analysis of Gal-DA1; and (C) MALDI-MS analysis of Gal-DA2.

Development solvent C:M:W = 130:50:9

GalNAcα1-O-NH_2_ + DHPA

GalNAcα1-O-N=DHPA

**Primulin staining**

(no orcinol response)


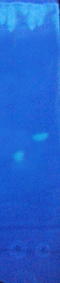


origin

solvent front

**Figure S3**: Analysis of the DHPA conjugation product of GalNAcα1-O-NH_2_.

**Figure S4.** Comparison of the binding signals of NGL products with single (DA1) and double (DA2) lipid chains immobilised covalently on nitrocellulose coated slides. The relative binding signals as fluorescence intensities are depicted as a heatmap for each of the lectins and antibodies used. The glycan sequence of each probe is referenced to as probe position listed in **Table S1**.

**Figure S5.** (A-H) Quality control of DA-NGL probes with *O*-GalNAc core sequences and different backbone and peripheral Fuc-, Gal-, Man- or GlcNAc- sequences using plant lectins and antibodies. The glycan sequence of probes eliciting binding signals are annotated. The representation of glycans follows the guidelines of Symbol Nomenclature for Glycans (Varki et al., 2015). The chart position assigned to each probe is referenced in **Table S1** (NGL Chart Pos).

**Figure S6. (**A-F**)** Comparison of glycan-binding patterns in non-covalent and covalent microarray platforms. The glycan sequence of probes eliciting binding signals are annotated. The representation of glycans follows the guidelines of Symbol Nomenclature for Glycans (Varki et al., 2015). The chart position assigned to each probe is referenced in **Table S1** (Covalent/NGL Chart Pos).

 **Figure S6 (cont.).** (G-L) Comparison of glycan-binding patterns in non-covalent and covalent microarray platforms.

**Figure S6 (cont.).** (M-N) Comparison of glycan-binding patterns in non-covalent and covalent microarray platforms.

**Table SI.** List of glycan probes in DA-NGL and covalent microarrays.

| **Probe Designation** | **Amino-Terminating Glycan and Control Probe Sequence^a^** | **Covalent/**  **NGL Chart Pos^b^** | **NGL Probes^c^** | **NGL**  **Chart**  **Pos^d^** | **Glycan Source^e^** |
| --- | --- | --- | --- | --- | --- |
| Amino-Terminating Glycan | | | | | |
| *Lactose-based* | | | | | |
| Lac-C2-N | Galβ-4Glcβ-O-C2-NH2 | 1 | -NX-DA | 1 | Present report |
| 2’FL-Gly | Fucα-2Galβ-4Glcβ-NH-Gly-NH2 | 2 | -NH-DA | 2 | Human milk (Likhosherstov et al. 2012) |
| B-tri-C3-N | Galα-3Galβ-O-C3-NH2  │  Fucα-2 | 3 | -NX-DA | 3 | Elicityl |
| 3’SA-Lac-C2-N | NeuAcα-3Galβ-4Glcβ-O-C2-NH2 | 4 | -NX-DA | 4 | Elicityl |
| 3’SA-Lac-Gly | NeuAcα-3Galβ-4Glcβ-NH-Gly-NH2 | 5 | -NH-DA | 5 | Human milk (Likhosherstov et al. 2016) |
| 6’SA-Lac-Gly | NeuAcα-6Galβ-4Glcβ-NH-Gly-NH2 | 6 | -NH-DA | 6 | Human milk (Likhosherstov et al. 2016) |
| *Lacto-N-tetraose and Lacto-N-neo-tetraose* | | | | | |
| LNFP I-Gly | Fucα-2Galβ-3GlcNAcβ-3Galβ-4Glcβ-NH-Gly-NH2 | 7 | -NH-DA | 7 | Human milk (Likhosherstov et al. 2012) |
| LSTb-Gly | Galβ-3GlcNAcβ-3Galβ-4Glcβ-NH-Gly-NH2  │  NeuAcα-6 | 8 | -NH-DA | 8 | Human milk (Likhosherstov et al. 2016) |
| DSLNT-Gly | NeuAcα-3Galß-3GlcNAcß-3Galß-4Glcß-NH-Gly-NH2  │  NeuAcα-6 | 9 | -NH-DA | 9 | Human milk (Likhosherstov et al. 2016) |
| DSMFLNH-Gly | Galß-4GlcNAcß-6   │ │   Fucα-3 Galß-4Glcβ-NH-Gly-NH2  │  NeuAcα-3Galß-3GlcNAcß-3  │  NeuAcα-6 | 10 | -NH-DA | 10 | Human milk (Likhosherstov et al. 2016) |
| *N-glycan* | | | | | |
| ChitoBiose-Gly | GlcNAcβ-4GlcNAcβ-NH-Gly-NH2 | 11 | -NH-DA | 11 | Chitin (Nishimura et al. 1989) |
| Fuc(3)GlcNAc-Gly | GlcNAcβ-NH-Gly-NH-DA  │ Fucα-3 | 12 | -NH-DA | 12 | Chemical synthesis (Likhosherstov et al. 2015) |
| Fuc(6)GlcNAc-Gly | Fucα-6GlcNAcβ-NH-Gly-NH2 | 13 | -NH-DA | 13 | Chemical synthesis (Likhosherstov et al. 2015) |
| DFuc(3,6)GlcNAc-Gly | Fucα-6GlcNAcβ-NH-Gly-NH2  │  Fucα-3 | 14 | -NH-DA | 14 | Chemical synthesis (Likhosherstov et al. 2015) |
| Man5-Gly | Manα-6  │ Manα-3Manα-6  │  Manβ-4GlcNAcβ-4GlcNAcβ-NH-Gly-NH2  │  Manα-3 | 15 | -NH-DA | 15 | Chicken egg white (Piskarev et al. 1990) |
| Man6-Gly | Manα-6  │ Manα-3Manα-6  │  Manβ-4GlcNAcβ-4GlcNAcβ-NH-Gly-NH2  │ Manα-2Manα-3 | 16 | -NH-DA | 16 | Chicken egg white(Piskarev et al. 1990) |
| NA2-Gly | Galβ-4GlcNAcβ-2Manα-6  │  Manβ-4GlcNAcβ-4GlcNAcβ-NH-Gly-NH2  │ Galβ-4GlcNAcβ-2Manα-3 | 17 | -NH-DA | 17 | Chicken egg white (Piskarev et al. 1990) |
| NA2F-Gly | Galβ-4GlcNAcβ-2Manα-6 Fucα-6  │ │  Manβ-4GlcNAcβ-4GlcNAcβ-NH-Gly-NH2  │ Galβ-4GlcNAcβ-2Manα-3 | - | -NH-DA | 18 | Chicken egg white (Piskarev et al. 1990) |
| *Glycolipid* | | | | | |
| SM1a-C3-N | Galβ-3GalNAcβ-4Galβ-4Glcβ-O-C3-NH2  │  SU-3 | 18 | -NH-DA | 19 | Present report |
| SM1a(2S)-C3-N | Galβ-3GalNAcβ-4Galβ-4Glcβ-O-C3-NH2  │  SU-2 | 19 | -NH-DA | 20 | Present report |
| *O-Glycan* | | | | | |
| GalNAcα-ON | GalNAcα-O-NH2 | 20 | -N=DA | 21 | Elicityl |
| GalNAcα-Ser | GalNAcα-O-Ser-NH2 | 21 | -NH-DA | 22 | Sussex Research |
| GalNAcα-Thr | GalNAcα-O-Thr-NH2 | 22 | -NH-DA | 23 | Dextra Lab |
| GalNAcβ-Ser | GalNAcβ-O-Ser-NH2 | - | -NH-DA | 24 | Present report |
| GalNAcβ-Thr | GalNAcβ-O-Thr-NH2 | - | -NH-DA | 25 | Present report |
| GlcNAcβ-Ser | GlcNAcβ-O-Ser-NH2 | - | -NH-DA | 26 | Present report |
| GlcNAcβ-Thr | GlcNAcβ-O-Thr-NH2 | - | -NH-DA | 27 | Present report |
| STn-Ser | NeuAcα-6GalNAcα-O-Ser-NH2 | 23 | -NH-DA | 28 | Present report |
| STn-Thr | NeuAcα-6GalNAcα-O-Thr-NH2 | 24 | -NH-DA | 29 | Present report |
| Core 1-Ser | Galβ-3GalNAcα-O-Ser-NH2 | 25 | -NH-DA | 30 | Dextra Lab |
| Core 1-Thr | Galβ-3GalNAcα-O-Thr-NH2 | 26 | -NH-DA | 31 | Sussex Research |
| SA1(2-3)-Core 1-Ser | NeuAcα-3Galβ-3GalNAcα-O-Ser-NH2 | 27 | -NH-DA | 32 | Present report |
| SA1(2-3)-Core 1-Thr | NeuAcα-3Galβ-3GalNAcα-O-Thr-NH2 | 28 | -NH-DA | 33 | Present report |
| SA1(2-6)-Core 1-Ser | Galβ-3GalNAcα-O-Ser-NH2  │  NeuAcα-6 | 29 | -NH-DA | 34 | Human urine(Parkkinen and Finne, 1983) |
| SA1(2-6)-Core 1-Thr | Galβ-3GalNAcα-O-Thr-NH2  │  NeuAcα-6 | 30 | -NH-DA | 35 | Human urine (Parkkinen and Finne, 1983) |
| SA2-Core 1-Ser | NeuAcα-3Galβ-3GalNAcα-O-Ser-NH2  │  NeuAcα-6 | - | -NH-DA | 36 | Human urine (Parkkinen and Finne, 1983) |
| SA2-Core 1-Thr | NeuAcα-3Galβ-3GalNAcα-O-Thr-NH2  │  NeuAcα-6 | 31 | -NH-DA | 37 | Human urine (Parkkinen and Finne, 1983) |
| Core 2-C3-N | Galβ-3GalNAcα-O-C3-NH2  │ GlcNAcβ-6 | 32 | -NH-DA | 38 | Nicolai Bovin |
| Core 2-Thr | Galβ-3GalNAcα-O-Thr-NH2  │ GlcNAcβ-6 | 33 | -NH-DA | 39 | Sussex Research |
| Core 3-Ser | GlcNAcβ-3GalNAcα-O-Ser-NH2 | 34 | -NH-DA | 40 | Sussex Research |
| Core 3-Thr | GlcNAcβ-3GalNAcα-O-Thr-NH2 | 35 | -NH-DA | 41 | Sussex Research |
| Gal-Core 3-Ser | Galβ-4GlcNAcβ-3GalNAcα-O-Ser-NH2 | 36 | -NH-DA | 42 | Present report |
| Gal(3S)-Core 3-Ser | Gal(3S)β-4GlcNAcβ-3GalNAcα-O-Ser-NH2 | 37 | -NH-DA | 43 | Present report |
| GalNAc-Gal(3S)-Core 3-Ser | GalNAcβ-4Gal(3S)β-4GlcNAcβ-3GalNAcα-O-Ser-NH2 | 38 | -NH-DA | 44 | Present report |
| GSC 967-Ser | Galβ-3GalNAcβ-4Gal(3S)β-4GlcNAcβ-3GalNAcα-O-Ser-NH2 | 39 | -NH-DA | 45 | Present report |
| Core 4-C3-N | GlcNAcβ-3GalNAcα-O-C3-NH2  │  GlcNAcβ-6 | 40 | -NH-DA | 46 | Nicolai Bovin |
| Core 4-Thr | GlcNAcβ-3GalNAcα-O-Thr-NH2  │  GlcNAcβ-6 | 41 | -NH-DA | 47 | Sussex Research |
| Man-PentaPep | YAT(Man)AV-NH2 | - | -NH-DA | 48 | Chemical synthesis (Bartels et al. 2016) |
| Man-HexaPep | CYAT(Man)AV-NH2 | - | -NMe-DA | 49 | Chemical synthesis (Bartels et al. 2016) |
| Man-UndecaPep | Sp-TriEG-SQSLEET(Man)ISPR-NH2 | - | -NH-DA | 50 | Chemical synthesis (Bartels et al. 2016) |
| *Glycosaminoglycan* | | | | | |
| Hep-4-NAc-PhN | GlcNAcα-4GlcAβ-4GlcNAcα-4GlcAβ-O-Ph-NH2 | - | -NH-DA | 51 | Chemo-enzymatic synthesis (Jian Liu) |
| Hep-4-NS-PhN | GlcNSα-4GlcAβ-4GlcNSα-4GlcAβ-O-Ph-NH2 | - | -NH-DA | 52 | Chemo-enzymatic synthesis (Jian Liu) |
| *Gluco-oligosaccharide* | | | | | |
| Glc12-C5-N | Glcβ-3Glcβ-3Glcβ-3Glcβ-[3Glcβ-3Glcβ]_3_-3Glcβ-3Glcβ-O-C5-NH2 | 42 | -NH-DA | 53 | Chemical synthesis (Weishaupt et al. 2013, 2017) |
| Glc15-C2-N | Glcβ-3Glcβ-3Glcβ-3Glcβ-[3Glcβ-3Glcβ]_3_-3Glcβ-3Glcβ-3Glcβ-3Glcβ-3Glcβ-O-C2-NH2 | 43 | -NH-DA | 54 | Chemical synthesis Novartis |
| Glc13(B10)-C5-N | Glcβ-3Glcβ-3Glcβ-3Glcβ-[3Glcβ-3Glcβ]_3_-3Glcβ-3Glcβ-O-C5-NH2  │  Glcβ-6 | 44 | -NH-DA | 55 | Chemical synthesis (Weishaupt et al. 2013, 2017) |
| *Monosaccharide* | | | | | |
| Gal-C2-N | Galβ-O-C2-NH2 | 45 | -NX-DA | 56 | Present report |
| Gal-Ph-N | Galβ-O-Ph-NH2 | - | -NH-DA | 57 | Present report |
| Glc-C2-N | Glcβ-O-C2-NH2 | 46 | -NX-DA | 58 | Present report |
| Man-C2-N | Manβ-O-C2-NH2 | - | -NH-DA | 59 | Present report |
| Xyl-C2-N | Xylβ-O-C2-NH2 | - | -NX-DA | 60 | Present report |
| Control Probes | | | | | |
| *Lactose- and N-acetyl lactosamine-based* | | | | | |
| Lac | Galβ-4Glc | - | -AO | 61 | (Palma et al. 2011; Vendele et al. 2020) |
| B-Tetra-T2 | Galα-3Galβ-4GlcNAc  │  Fucα-2 | - | -AO | 62 | (Vendele et al. 2020) |
| NeuAcα-(3')Lac | NeuAcα-3Galβ-4Glc | - | -AO | 63 | (Palma et al. 2011; Vendele et al. 2020) |
| *Lacto-N-tetraose and Lacto-N-neo-tetraose* | | | | | |
| LNFP-I | Fucα-2Galβ-3GlcNAcβ-3Galβ-4Glc | - | -AO | 64 | (Palma et al. 2011; Vendele et al. 2020) |
| LNFP-III | Galβ-4GlcNAcβ-3Galβ-4Glc  │  Fucα-3 | - | -DH | 65 | (Palma et al. 2011; Vendele et al. 2020) |
| LSTb | Galβ-3GlcNAcβ-3Galβ-4Glc  │ NeuAcα-6 | - | -DH | 66 | (Palma et al. 2011; Vendele et al. 2020) |
| *Poly N-acetyl lactosamine* | | | | | |
| H2 (with H2+Fuc)* | Fucα-2Galβ-4GlcNAcβ-3Galβ-4GlcNAcβ-3Galβ-4Glcβ-Cer | - | - | 67 | (Gao et al. 2014) |
| TFiLNO | Galβ-3GlcNAcβ-3Galβ-4GlcNAcβ-6  │ │ │  Fucα-4 Fucα-3 Galβ-4Glc  │  Galβ-3GlcNAcβ-3  │  Fucα-4 | - | -DH | 68 | (Palma et al. 2011; Vendele et al. 2020) |
| *Glycolipid* | | | | | |
| SM1a | Galβ-3GalNAcβ-4Galβ-4Glcβ-Cer  │  SU-3 | - | - | 69 | (Palma et al. 2011; Vendele et al. 2020) |
| Asialo-GM1-Tetra | Galβ-3GalNAcβ-4Galβ-4Glc | - | -DH | 70 | (Palma et al. 2011; Vendele et al. 2020) |
| SB1a | SU-3Galβ-3GalNAcβ-4Galβ-4Glcβ-Cer  │  SU-3 | - | - | 71 | (Palma et al. 2011; Vendele et al. 2020) |
| *O-Glycan* | | | | | |
| GalNAc-Ser | GalNAcα-Ser | - | -DH | 72 | (Palma et al. 2011; Vendele et al. 2020) |
| GalNAc-Thr | GalNAcα-Thr | - | -DH | 73 | (Palma et al. 2011; Vendele et al. 2020) |
| BSM-Di-A1 | NeuGcα-6GalNAc | - | -AO | 74 | (Palma et al. 2011; Vendele et al. 2020) |
| BSM-Di-A2 | NeuAcα-6GalNAc | - | -AO | 75 | (Palma et al. 2011; Vendele et al. 2020) |
| Galβ-3GalNAc | Galβ-3GalNAc | - | -AO | 76 | (Palma et al. 2011; Vendele et al. 2020) |
| Galβ-6GalNAc | Galβ-6GalNAc | - | -AO | 77 | (Palma et al. 2011; Vendele et al. 2020) |
| DST | NeuAcα-3Galβ-3GalNAc  │  NeuAcα-6 | - | -AO | 78 | (Palma et al. 2011; Vendele et al. 2020) |
| *Gluco-oligosaccharide* | | | | | |
| Curd-13 | Glcβ-3Glcβ-3Glcβ-3Glcβ-[3Glcβ-3Glcβ]_3_-3Glcβ-3Glcβ-3Glc | - | -AO | 79 | (Palma et al. 2015) |
| Cello-3 | Glcβ-4Glcβ-4Glc | - | -AO | 80 | (Palma et al. 2015) |
| *N-Acetylglucosamine oligomer* | | | | | |
| GN2 | GlcNAcβ-4GlcNAc | - | -AO | 81 | (Palma et al. 2011; Vendele et al. 2020) |
| GN6 | GlcNAcβ-4GlcNAcβ-4GlcNAcβ-4GlcNAcβ-4GlcNAcβ-4GlcNAc | - | -AO | 82 | (Vendele et al. 2020) |

^a^ Abbreviations of reducing terminal structures. For the amino-terminating glycans: C2, -CH_2_-CH_2_-; C3, -CH_2_-CH_2_-CH_2_-; C5, -CH_2_-CH_2_-CH_2_-CH_2_-CH_2_-; Gly, glycine; Ser, serine; Thr. threonine; Ph, phenyl. Reducing oligosaccharides and glycosylceramides were used as reference glycans; Cer, ceramide.

^b^ The chart positions of glycan probes in the comparison of the DA-NGL arrays and covalent arrays (**Figure 5** and **Figure S6**).

^c^ The reducing terminal structures of the DA-NGLs with single lipid prepared using the aldehyde-terminating lipid reagent with the amino-terminting glycans; -NX: NH or NMe. For the control probes: DH, NGLs prepared from reducing glycans by reductive amination with the amino lipid DHPE; AO, NGLs prepared from reducing glycans by oxime ligation with aminooxy (AO) functionalized DHPE.

^d^ The chart positions assigned to glycan probes in the analysis of the DA-NGL arrays (**Figures 3-5** and **Figures S4 and S5**).

^e^ References or sources are given for the amino-terminating glycans.

**Table SII.** List of proteins investigated and their reported glycan recognition, sources and summarised information on the assay conditions and detection systems used for their analysis.

| **Proteins** | **Source^a^** | **Analysis concentration** | **Detection Antibodies/ concentration** | **Reported Glycan recognition^b^** |
| --- | --- | --- | --- | --- |
| *Vicia Villosa* lectin (VVL/VVA) | Vector B-1235 (Biotinylated) | 50 µg/ml | NA | α-*N*-acetylgalactosamine (α-GalNAc)(Godula and Bertozzi, 2012; Puri et al. 1992) |
| *Helix pomatia* lectin  (HPA) | Sigma L-6512  (Biotinylated) | 50 µg/ml | NA | Terminal α-GalNAc residues  GalNAcα3Gal, β-GalNAc and GlcNAcβ4Gal(Godula and Bertozzi, 2012; Sanchez et al. 2006) |
| Peanut agglutinin  (PNA) | Vector B-1075  (Biotinylated) | 50 µg/ml | NA | Galβ3GalNAc (core 1) and Galβ1-3(GlcNAcβ1-6)GalNAc (core 2) (CFG glycan array)^c^ |
| *Aleuria aurantia* lectin  (AAL) | Vector B-1395  (Biotinylated) | 2 µg/ml | NA | Fucose-terminating oligosaccharides (Fucα2Galβ4/3(Fucα3/4)Galβ4GlcNAc);  Core α6-, α3-fucosylated *N*-glycans (Gao et al. 2014) |
| *Ulex europeus* agglutinin (UEA-1) | Vector B-1065 (Biotinylated) | 50 µg/ml | NA | H-type 2 antigen (Fucα2Galβ4GlcNAc-) (Gao et al. 2014) |
| *Ricinus Communis* agglutinin I (RCA_120_) | Vector B-1085  (Biotinylated) | 5 µg/ml | NA | Non-reducing Galβ-linked oligosaccharides |
| Concanavalin A  (ConA) | Vector B-1005  (Biotinylated) | 2 µg/ml | NA | Manα-linked oligosaccharides; high-mannose *N*-glycans |
| Wheat Germ Agglutinin (WGA) | Vector B-1025  (Biotinylated) | 5 µg/ml | NA | Nonreducing GlcNAcβ4-terminating oligosaccharides  Weak binding to 2-3/2-6 sialylated oligosaccharides |
| CTD110.6 – Anti-O-GlcNAc (IgM kappa light chain) | Santa Cruz sc-59623 | 2 µg/ml | Biotinylated anti-Mouse IgG (whole molecule)  (SIGMA B-7264); 10 µg/ml | β-*O*-GlcNAc(Comer et al., 2001) |
| Anti-Sialyl Tn  Clone 3F1 (IgG) | SBH Biosciences -ASTn | 10 µg/ml | Biotinylated anti-Mouse IgG (whole molecule)  (SIGMA B-7264); 10 µg/ml | NeuAcα2-6GalNAc- and NeuGcα2-6GalNAc- (CFG glycan array)^d^ |
| Anti-blood group H-type 1 [17-206] | Abcam ab3355 | 1/50 | Biotinylated anti-Mouse IgG (whole molecule)  (SIGMA B-7264); 1/200 | H-type 1 antigen (Fucα2Gaβ3GlcNAc-) (Gao et al. 2014) |
| Anti-blood group H-type 2  [BRIC231] | Abcam ab33404 | 10 µg/ml | Biotinylated anti-Mouse IgG (whole molecule)  (SIGMA B-7264);10 µg/ml | H-type 2 antigen(Gao et al., 2014)  Fucα2Gaβ4GlcNAc- |
| *Cm*CBM6-2  *Cellvibrio mixtus*  Endoglucanase 5A; CAZY family 6 | Recombinant protein domain prepared with an N- terminal hexa-histidine-tag(Henshaw et al. 2004) | 10 µg/ml;  Pre-complexed with detection antibodies, ratio 1:3:3 by weight | Ab1: Mouse monoclonal anti-poly-Histidine  (SIGMA H1029); 30 µg/ml  Ab2: Biotinylated anti-Mouse IgG (whole molecule) (SIGMA B-7264); 30 µg/ml | Type C carbohydrate binding module (CBM)  Two independent glucan binding sites: cleft A and B  Broad specificity to β-linked glucose oligosaccharides (Henshaw et al. 2004); (Palma et al. 2015) |
| *Tm*CBM4-2  *Thermotogoa maritime* laminarinase  CAZY family 4 | Recombinant protein domain prepared with an N- terminal hexa-histidine-tagged(Boraston et al. 2001) | 10 µg/ml;  Pre-complexed with detection antibodies, ratio 1:3:3 by weight | Ab1: Mouse monoclonal anti-poly-Histidine (SIGMA H1029); 30 µg/ml  Ab2: Biotinylated anti-Mouse IgG (whole molecule) (SIGMA B-7264); 30 µg/ml | Type B CBM highly specific for linear β3-linked gluco-oligosaccharides >DP- 2, peaks at DP-5; binding site is a deep groove, requiring the internal oligosaccharide sequence(Boraston et al. 2001); (Palma et al. 2015) |
| Macrophage galactose-type lectin (MGL) | Abcam (human Fc-tagged) ab219892 | 10 µg/ml  Pre-complexed with detection antibody, ratio 1:1 by weight | Biotinylated anti-human IgG (whole molecule) (Vector BA3000); 10 µg/ml | GalNAcα-Ser/Thr (Marcelo et al. 2014)  NeuAc2-6GalNAcα-Ser/Thr (Sialyl-Tn antigen) (Mortezai et al. 2013) |
| Murine Dectin-1 | Sino Biological  (hexa-histidine-tagged) 50233-M07H | 30 µg/ml | Pre-complex of Ab1 and Ab2  Ab1: Anti-poly-Histidine (SIGMA H1029); 10 µg/ml  Ab2: Biotinylated anti-Mouse IgG (whole molecule) (SIGMA B-7264); 10 µg/ml | Linear β1,3 gluco-oligosaccharides, DP-10 and longer (microarray analysis)(Palma et al. 2006, 2015)  β1,3-linked linear gluco-octasaccharide with a β1,6-linked mono-glucosyl branch (SPR/inhibition of binding to glucanphosphate)(Adams et al. 2008)  β1,3-linked linear gluco-hexadecasaccharide with or without a β1,6-linked mono-glucosyl branch(Tanaka et al. 2012) |
| Human Siglec-15 | R&D systems (human Fc-tagged) 9227-SL | 2 µg/ml,  Pre-complexed with detection antibody, ratio 1:1 by weight;  20 µg/ml,  Non pre-complexed | Biotinylated anti-human IgG (whole molecule) (Vector BA3000); 2 µg/ml | NeuAc2-6GalNAcα- (linked to PAA) (Takamiya et al. 2013) |
| P[19]VP8* | Recombinant protein domain prepared as GST-tagged (Li et al. 2018) | 50 µg/ml | Anti-GST (SANTA CRUZ GST Z-5);1/200  Biotinylated anti-rabbit IgG (SIGMA B-7389)); 1/200 | Mucin core 2, 4 and 6 sequences(Liu et al., 2016)  Blood group H type 1 sequences; non-fucosylated type 1 backbone sequences (Li et al. 2018) |

^a^ The commercial source or the reference to the preparation of the recombinant proteins is indicated.

^b^ The main glycan-binding is indicated with a reference to a particular study.

^c^ CFG glycan array at http://www.functionalglycomics.org/glycomics/HServlet?operation=view&sideMenu=no&psId=primscreen_4678.

^d^ CFG glycan array at http://www.functionalglycomics.org/glycomics/search/jsp/result.jsp?query=primscreen_1404).

**Table SIII.** Supplementary glycan microarray document based on MIRAGE guidelines (doi:[10.3762/mirage.3](http://www.beilstein-institut.de/en/projects/mirage/guidelines#glycan_microarrays)).

| **Classification** | **Guidelines** |
| --- | --- |
| 1. **Sample: Glycan Binding Sample** | |
| Description of Sample | Sample names:  Described in **Table S2**. |
| Sample modifications | Not relevant. |
| Assay protocol | Described in the **Methods Section**. |
| **2.** **Glycan Library** | |
| Glycan description for defined glycans | Sixty amino-terminating (including aminoalkyl-, phenyl- or glycine-terminating, or natural serine or threonine amino acids) glycans were used in this study together with reference NGLs, prepared from reducing oligosaccharides, and glycosylceramides. These are listed in **Table S1**. |
| Glycan description for undefined glycans | Not relevant. |
| Glycan modifications | For the covalent microarrays, the amino-terminating glycans were used without modification.  For non-covalent microarrays, the amino-terminating glycans were conjugated to the new aldehyde-functionalized phospholipid reagent N-(4-formylbenzamide)-1,2-dihexadecyl-sn-glycero-3-phosphoethanolamine (DHPA) by reductive amination as described in **Methods Section**. Reference NGLs of reducing oligosaccharides were prepared by reductive amination with the amino lipid, 1,2-dihexadecyl-*sn*-glycero-3-phosphoethanolamine [(DHPE)(Chai et al. 2003) or by oxime ligation with an aminooxy-functionalized DHPE [(AOPE)(Liu et al. 2007). These are listed in **Table S1**. |
| 1. **3.** **Printing Surface; e.g., Microarray Slide** | |
| Description of surface | 16-pad nitrocellulose-coated glass microarray slides;  16-pad NHS-functionalized glass microarray slides. |
| Manufacturer | UniSart® 3D Microarray Slide Sartorius (Goettingen, Germany);  Schott Nexterion H (Jena, Germany). |
| Custom preparation of surface | Not relevant. |
| Non-covalent Immobilisation | The lipid-linked oligosaccharide probes were arrayed on nitrocellulose-coated glass slides as liposomes in the presence of the carrier lipids, 1,2-di-O-hexadecyl-*sn*-glycero-3-phosphocholine (DHPC) and cholesterol (Liu et al. 2012). |
| Covalent Immobilisation | The amino-teminating glycans were diluted in 100 mM phosphate buffer, pH 8.7 and printed onto NHS-functionalized glass slides. |
| **4. Arrayer (Printer)** | |
| Description of Arrayer | Nano-Plotter 2.1 (GeSiM, Radeberg, Germany). |
| Dispensing mechanism | Non-contact liquid delivery with four dispensing tips. |
| Glycan deposition | Approximately 0.33 nl was printed per spot. |
| Printing conditions non-covalent microarray | The printing solutions were aqueous based. Printing was performed at ambient temperature and relative humidity of 60%. The NGL printing solutions contained 100 µM of DHPC and cholesterol (both from SIGMA) as lipid carriers in addition to the lipid-linked glycan probes. The concentrations of the NGL probes were 5 and 15 µM, for the 2 and 5 fmol per spot levels, respectively.  The printing solutions also contained Cyanine 3 NHS ester (GE Healthcare) at 20 ng/ml (26 nM) as a tracer for quality control of arraying process and for localization of the printed spots. |
| Printing conditions covalent microarray | The probes were printed at a concentration of 100 µM in the printing buffer (100 mM phosphate buffer, pH 8.7). As a grid alignment control Alexa Fluor 647-labeled streptavidin (100 µM in 100 µg/ml BSA in printing buffer) was used. Printing was carried out at 60% relative humidity, followed by probe immobilization overnight on the arrayer slide deck at 80% relative humidity in the dark. The remaining reactive NHS groups were blocked for 2h with 50 mM ethanolamine in 100 mM borate buffer pH 8.7. The slides were rinsed 3 times with 100 mM Phosphate Buffer Saline pH 7.4 with 0.05% Tween 20, followed by rinsing with water. |
| 1. **5.** **Glycan Microarray with “Map”** | |
| Array layout | The arrayed slides contained 16 identical pads (subarrays). Each subarray was set up for printing 64 probes maximum, each at 2 levels in duplicate (non-covalent arrays) or one level in quadruplicate (covalent arrays) (four spots for one probe in a row); 256 spots (16x16) in total for 64 probes. |
| Glycan identification and quality control | Quality control of the DA-NGL non-covalent and covalent microarrays was carried out with the proteins listed in **Table S2**. |
| 1. **6. Detector and Data Processing** | |
| Scanning hardware | GenePix 4300A (Molecular Devices). |
| Scanner settings non-covalent microarray | Scanning resolution: 10 μm / pixel (this resolution is adequate for the sizes of sample spots);  Laser channel: Red (wavelength 635 nm);  Laser settings: PMT: 350; Scan powers: 10%, 20% or 90% to achieve maximum signal without spot saturation. |
| Scanner settings covalent microarray | Scanning resolution: 5 μm / pixel (this resolution is adequate for the sizes of sample spots)  Laser channel: Red (wavelength 635 nm)  Laser settings: PMT: 450; scan power: 100% to achieve maximum signal without spot saturation. |
| Image analysis software | GenePix® Pro 7 (Molecular Devices). |
| Data processing | Spots were defined as circular features with a variable radius as determined by the Genepix scanning software. Local background subtraction was performed. The resulting gpr file was entered into an in-house microarray database using software (designed by Mark Stoll, <http://www.beilstein-institut.de/en/publications/proceedings/glyco-2009>) for data processing. No particular normalisation method or statistical analysis was used. |
| **7.** **Glycan Microarray Data Presentation** | |
| Data presentation | The microarray binding results are in **Figs. 3-6, Figures S4-S6**. |
| 1. **8.** **Interpretation and** **Conclusion from Microarray Data** | |
| Data interpretation | For plotting and comparing binding intensities the charts and heatmaps in different analyses the software (<http://www.beilstein-institut.de/en/publications/proceedings/glyco-2009>) developed by Mark Stoll was used. |
| Conclusions | Here we describe a new (DA-NGL) microarray prepared from amino-terminating glycans for detecting binding patterns with carbohydrate-recognizing proteins and its use to extend the repertoire of glycan libraries and to compare covalent and non-covalent microarray platforms. The microarray analyses showed that the two types of microarrays give concordant binding profiles and specificity for the different classes of glycan binding proteins investigated, with differences being observed in detection of weak binders. The integrated information from the two microarrays has provided additional insights into the identification of a range sialylated glycan ligands for Siglec-15 in addition to the sialyl-Tn-Ser/Th, the specificity of Dectin-1 towards linear rather than branched β1-3-glucans, and to the preferential recognition of core-2 and core-4 among the *O*-glycan cores and blood group H type 1 pentasaccharide antigens by the adhesive protein VP8* of the rotavirus P[19]. |

**Supplementary methods**

**Syntheses of Gal-C2-NH_2_, Glc-C2-NH_2_, Man-C2-NH_2_, Xyl-C2-NH_2_, Lac-C2-NH_2_ and Gal-PhNH_2_**

Amino-terminating mono- and disaccharides were prepared from corresponding monosaccharide or disaccharide by a published method (Šardzík et al. 2010) as described in **Supplementary Scheme 1** using Gal-C2-NH_2_ as the example to obtain Gal-C2-NH2 (**6**) , Glc-C2-NH2 (**7**) , Man-C2-NH2 (**8**), Xyl-C2-NH2 (**9**) and Lac-C2-NH2 (**10**).

**Supplementary Scheme 1**. Synthesis of Gal-C2-NH2 (**6**) , Glc-C2-NH2 (**7**) , Man-C2-NH2 (**8**), Xyl-C2-NH2 (**9**) and Lac-C2-NH2 (**10**) : a) Ac_2_O, AcONa, 120 ºC, 52%; b) HOCH_2_CH_2_Br, BF_3_·Et_2_O, DCM, 0 ºC-r.t., 76%; c) NaN_3_, DMF, 80 ºC, overnight, 94%; d) CH_3_ONa/CH_3_OH, pH 8-9; e) 10% Pd/C, H_2_, 18 h, 92% for two steps.

Gal-PhNH_2_ (**13**) was prepared from penta-*O*-acetyl-β-D-galacotose by a three-step route (**Supplementary** **Scheme 2**).

**Supplementary Scheme 2**. Synthesis of Gal-PhNH_2_ (**13**) : a) *p*-Nitrophenol, BF_3_·Et_2_O, 4 Å MS, DCM, 0ºC-r.t., 70%; b) CH_3_ONa/CH_3_OH, pH8-9; c) 10% Pd/C, H_2_, 24 h, 86% for two steps.

*2'-Azidoethyl-2,3,4,6-tetra-O-acetyl-β-D-galactopyranoside* (**4**) (Yarlagadda et al. 2015) *−* Compound **2** (Michihata et al. 2013) (10 g, 25.6 mmol) and 2-bromoethanol (2.4 ml, 33.3 mmol) were dissolved in 50 ml dry DCM, and the solution was cooled in an ice bath. BF_3_·Et_2_O (8.4 ml, 33.3 mmol) was added. The reaction mixture was stirred under nitrogen at 0^o^C for 0.5 h, and then at r.t. for 6 h. The mixture was washed sequentially with saturated NaHCO_3_ solution (3×60 mL), water (2×60 mL), and brine (2×60 ml), dried with Na_2_SO_4_, and concentrated. The residue was purified by column chromatography on silica gel (petroleum ether/EtOAc, 2.5:1~1.5:1) to give **3** (8.86 g, 76%).

Compound **3** (4.00 g, 8.8 mmol) was dissolved in 70 ml DMF, and NaN_3_ (4.00 g, 61.6 mmol) was added. The reaction mixture was stirred at 80^o^C overnight. The mixture was filtered and evaporated. The residue was diluted with ethyl acetate, and this mixture was washed with water (3×30 ml), and brine (2×30 ml), dried with Na_2_SO_4_, and concentrated and dried to give compound **4** (3.44 g, 94%). ^1^H NMR (500 MHz, CDCl_3_): *δ* 5.40 (dd, *J* = 2.7, 1.0 Hz,1H, H-4), 5.25 (dd, *J* = 8.0, 10.4 Hz, 1H, H-2), 5.03 (dd, *J* = 3.4, 10.5 Hz, 1H, H-3), 4.57 (d, *J* = 8.0 Hz, 1H, H-1), 4.19 (dd, *J* = 6.6, 11.3 Hz, H-6b), 4.13 (dd, *J* = 6.8, 11.3 Hz, 1H, H-6a), 4.05 (ddd, *J* = 3.7, 4.6, 10.7Hz, 1H, H-5), 3.70 – 3.93(m, 2H, -OC*H*_2_CH_2_N_3_), 3.31 – 3.51 (m, 2H,-OCH_2_C*H*_2_N_3_), 2.16 (s, 3H, -OCOC*H*_3_), 2.07 (s, 3H, -OCOC*H*_3_), 2.05 (s, 3H, -OCOC*H*_3_), 1.99 (s, 3H,-OCOC*H*_3_). ESI-MS: *m/z* calculated for C_16_H_23_NaN_3_O_10_ [M+Na]^+^ 440.1, found 440.0.

*2'-Aminoethyl-β-D-galactopyranoside* (**6**) (Šardzík et al. 2010; Yarlagadda et al. 2015) *−* Compound **4** (1.08 g, 2.59 mmol) was dissolved in 30 ml MeOH, and a solution of NaOMe (0.5 *M* in MeOH) was added to obtain a basic pH (8–9, pH paper). The mixture was stirred for 30 min, and then it was neutralized with ion-exchange resin (H^+^). The resin was removed by filtration, and the solvent was concentrated to give compound **5**.

Then 10% Pd/C (500 mg) was added to a solution of compound **5** in methanol (15.0 mL). The mixture was stirred at room temperature under a hydrogen atmosphere (balloon) for 24 h. The mixture was filtered through Celite, and the filtrate was concentrated in vacuo to give compound **6** (530 mg, 92% for two steps). ^1^H NMR (500 MHz, CD_3_OD): *δ* 4.30 (d, *J* = 7.6 Hz, 1H, H-1), 4.09 – 3.97 (m, 1H,-O*CH_2_*CH_2_NH_2_), 3.96 – 3.86 (m, 1H, -O*CH_2_*CH_2_NH_2_,), 3.83 (d, *J* = 3.0 Hz, 1H, H-4), 3.75 (dd, *J* = 11.4, 6.1 Hz, 2H, H-6), 3.59 – 3.52 (m, 2H, H-2, H-5), 3.49 (dd, *J* = 9.7, 3.2 Hz, 1H, H-3), 3.24 – 3.08 (m, 2H, -OCH_2_*CH_2_*NH_2_). ESI-MS: *m/z* calculated for C_8_H_18_NO_6_ [M+H]^+^ 224.1, found 224.1.

Glc-C2-NH_2_ Man- C2-NH_2_, Xyl- C2-NH_2_ and Lac-C2-NH_2_ were similarly prepared and analyzed with the following analytical data.

Glc-C2-NH2 (Šardzík et al. 2010) (**7**): ^1^H NMR (500 MHz, CD_3_OD): *δ* 4.26 (d, *J* = 7.8 Hz, 1H, H-1), 4.04 – 3.60 (m, 6H), 3.40 – 3.33 (m, 2H, -O*CH_2_*CH_2_NH_2_), 2.88 – 2.74 (m, 2H, -OCH_2_*CH_2_*NH_2_). ESI-MS: calculated for C_8_H_18_NO_6_[M+H]^+^ 224.1, found m/z 224.1.

Man- C2-NH2 (Šardzík et al. 2010) (**8**): ^1^H NMR (500 MHz, CD_3_OD): *δ* 4.72 (d, *J* = 2.4 Hz, 1H, H-1), 3.88 – 3.54 (m, 6H), 3.53 – 3.46 (m, 2H, -O*CH_2_*CH_2_NH_2_), 2.85 – 2.68 (m, 2H, -OCH_2_*CH_2_*NH_2_). ESI-MS: *m/z* calcd for C_8_H_18_NO_6_[M+H]^+^ 224.1, found 224.2.

Xyl- C2-NH2 (Šardzík et al. 2010) (**9**): ^1^H NMR (500 MHz, CD_3_OD): *δ* 4.17 (d, *J* = 7.5 Hz, 1H, H-1), 3.91 (dd, *J* = 10.3, 4.7 Hz, 1H, H-5a), 3.86 – 3.79 (m, 1H, -O*CH_2_*CH_2_NH_2_), 3.66 – 3.59 (m, 1H, -O*CH_2_*CH_2_NH_2_), 3.48 – 3.41 (m, 1H, H-4), 3.27 – 3.13 (m, 4H, H-3, H-2, H-5b, -OCH_2_*CH_2_*NH_2_), 3.10 – 3.05 (m, 1H, -OCH_2_*CH_2_*NH_2_). ESI-MS: *m/z* calculated for C_7_H_16_NO_5_ [M+H]^+^ 194.1, found 194.1.

Lac- C2-NH2 (Šardzík et al. 2010) (**10**): ^1^H NMR (500 MHz, CD_3_OD): δ 4.55 (d, 1H, *J* = 7.6 Hz), 4.51 (d, 1H, *J* = 7.8 Hz), 3.94 – 3.87 (m, 2H), 3.84(d, 1H, *J* = 4.2 Hz), 3.82 – 3.79 (m, 1H), 3.78 – 3.74 (m, 1H), 3.69 (dd, *J* = 11.4, 4.6 Hz, 1H), 3.66 – 3.60(m, 1H), 3.60 – 3.50 (m, 4H), 3.48 (dd, *J* = 9.7, 3.2 Hz, 1H), 3.44 – 3.38 (m, 1H), 3.55 – 3.52 (m, 1H), 2.90 – 2.80 (m, 2H). ESI-MS: *m/z* calculated for C_14_H_28_NO_11_ [M＋H]^＋^386.4, found 386.1.

*p-Nitrophenyl-2,3,4,6-tetra-O-acetyl-β-D-galactopyranoside* (Kumar et al. 2014) (**11**) *−* A suspension of compound **2** (1.00 g, 2.56 mmol), *p*-nitrophenol (530 mg, 3.84 mmol), and molecular sieves (4Å; 1.50 g) in 30 ml dry DCM was stirred for 0.5 h, and then cooled to 0 °C. BF_3_·Et_2_O (0.97 ml, 3.84 mmol) was added, and the mixture was stirred under nitrogen for 0.5 h, and then stirred over night at room temperature. The mixture was filtered through Celite. The filtrate was washed with saturated NaHCO_3_ solution (3×30 mL), and brine (2×30 mL), dried with Na_2_SO_4_, and concentrated. The residue was purified by column chromatography on silica gel (petroleum ether/EtOAc, 3:1~1.5:1) to give **11** (841 mg, 70%).^1^H NMR (500 MHz, CDCl_3_): *δ* 8.23-8.18 (m, 2H, Ph-*H*), 7.11-7.05 (m, 2H, Ph-*H*), 5.52 (dd, *J* = 10.4, 7.9 Hz, 1H, H-2), 5.48 (dd, *J* = 3.3, 1.0 Hz, 1H, H-4), 5.17 (d, *J* = 7.9 Hz, 1H, H-1), 5.13 (dd, *J* = 10.4, 3.4 Hz, 1H, H-3), 4.22 (dd, *J* = 11.0, 6.9 Hz, 1H, H-6a), 4.18 – 4.14 (m, 1H, H-5), 4.13 (dd, *J* = 10.8, 4.0 Hz, 1H, H-6b), 2.19 (s, 3H, C*H*_3_CO), 2.07 (s, 6H, 2×C*H*_3_CO), 2.02 (s, 3H, C*H*_3_CO); ^13^C NMR (125 MHz, CDCl_3_): δ 170.25, 170.07, 170.00, 169.22, 161.17, 143.21, 125.77, 116.56, 98.60, 71.46, 70.56, 68.26, 66.66, 61.32, 20.67, 20.64, 20.61, 20.53. ESI-MS: *m/z* calculated for C_20_H_23_NNaO_12_ [M+Na]^+^492.1, found 492.2.

*4′-aminophenyl-β-D-galactopyranoside* (Capicciotti et al. 2016) (**13**) *−* Compound **11** (50 mg, 0.106 mmol) was dissolved in MeOH, and a solution of NaOMe (0.5 *M* in MeOH) was added to obtain a basic pH (8–9). The mixture was stirred for 30 min, and then it was neutralized with ion-exchange resin (H^+^). The resin was removed by filtration, and the solvent was concentrated to give **12**. 10% Pd/C (50 mg) was added to a solution of compound **13** in methanol (5.0 mL). The mixture was stirred at room temperature under a hydrogen atmosphere (balloon) for 24 h. The mixture was filtered through Celite, and the filtrate was concentrated in vacuo to give compound **13** (25 mg, 86%). ^1^H NMR (500 MHz, CD_3_OD): *δ* 7.00 – 6.91 (m, 2H, Ph-*H*), 6.71 – 6.57 (m, 2H, Ph-*H*), 4.68 (d, *J* = 7.7 Hz, 1H, H-1), 3.88 (d, *J* = 3.2 Hz, 1H, H-4), 3.77 – 3.73 (m, 3H, H-6a, H-2, H-5), 3.61 (dd, *J* = 13.7, 7.9 Hz, 1H, H-6b), 3.54 (dd, *J* = 9.6, 3.4 Hz, 1H, H-3). ESI-MS: *m/z* calculated for C_12_H_18_NO_6_ [M+H]^+^ 272.3, found 272.2.

**Synthesis of SM1a-C3-NH_2_ and SM1a(2S)-C3-NH_2_**

The sulfated ganglioside SM1a has been synthesized as described (Zhang et al. 2015). Two amino-terminating SM1a analogs were prepared as follows. SM1a-C3-NH_2_ was obtained from a trisaccharide donor **17** (Zhang et al., 2015) and an acceptor **16.** Scheme and methods for the preparation of key intermediates and **SM1a** analogs are illustrated below.

**Supplementary Scheme 3.** Synthesis of SM1a-C3-NH_2_ (**22**) and SM1a(2S)-C3-NH2 (**23**): a) NIS, TfOH, 4Å MS, DCM, 0^o^C, 44%; b) i. AcOH, H_2_O, 80^o^C; ii. BBTZ, DCM, Et_3_N, 77%; c) TMSOTf, AW300, DCM, 0^o^C, 85%; d) TBAF, AcOH, THF, 0^o^C to rt, 92% for **19**; TBAF, THF, 0^o^C to rt, 90% for **19a**; e) SO_3_⋅Py, DMF, 90^o^C, 95% for **20**, 92% for **20a**; f) NaOMe, MeOH/THF, 95% for **21**, 97% for **21a**; g) 10% Pd/C, H_2_, MeOH, 87% for **22**, 99% for **23**.

*3-Azidopropyl-2,3-di-O-benzoyl-4,6-O-benzylidene-β-D–glucopyranoside* (**15**) (Lefeber et al. 2001) *−* A suspension of compound **14** (Lin et al. 2000) 100 mg, 0.17 mmol), 3-azido-1-propanol (25 mg, 0.34 mmol) and molecular sieves (4Å, 200 mg) in CH_2_Cl_2_ (5.0 mL) was stirred for 1 h, and then cooled to 0°C. To the mixture were added *N*-iodosuccinimide (NIS; 76 mg, 0.34 mmol) and trifluoromethanesulfonic acid (TfOH, 4.5 μl, 0.051 mmol) and stirring was continued for 0.5 h. Completion of the reaction was confirmed by TLC (petroleum ether/EtOAc, 3.5:1). Et_3_N was added to quench the reaction. The mixture was filtered through Celite. The combined filtrate was washed with saturated Na_2_S_2_O_3_ (2×20 ml), satd NaHCO_3_ (2×20 ml), and brine (2×20 ml), dried over Na_2_SO_4_, and concentrated. The residue was purified by column chromatography on silica gel (petroleum ether/EtOAc, 7:1) to give **15** (42 mg, 44%). ^1^H NMR (600 MHz, CDCl_3_): *δ* 7.98 – 7.30 (m, 15H, Ar*H*), 5.79 (t, *J* = 9.6 Hz, 1H, H-3), 5.55 (s, 1H, PhC*H*), 5.47 (dd, *J* = 9.5, 7.9 Hz, 1H, H-2), 4.79 (d, *J* = 7.8 Hz, 1H, H-1), 4.44 (dd, *J* = 10.6, 4.9 Hz, 1H, H-4), 4.01 – 3.97 (m, 1H, H-α), 3.93 (t, *J* = 9.5 Hz, 1H, H-6a), 3.89 (t, *J* = 10.3 Hz, 1H, H-6b), 3.71 (td, *J* = 9.7, 5.0 Hz, 1H，H-5), 3.62 (ddd, *J* = 9.9, 7.7, 4.8 Hz, 1H, H-α), 3.29-3.19 (m, 2H, H-γ), 1.85 – 1.71 (m, 2H, H-β).

*3-Azidopropyl-2,3,6-tri-O-benzoyl-β-D–glucopyranoside* (**16**) (Lefeber et al. 2001)  *−* To a solution of AcOH/H_2_O (9:1, 5 ml), **15** (38 mg, 0.070 mmol) was added. The solution was stirred at 80°C for 2 h. The solution was concentrated, and 5 mL toluene was added to co-evaporate once. The residue was dissolved with 5 mL CH_2_Cl_2_, then Et_3_N (12 μl, 0.0815 mmol) and BBTZ (19 mg, 0.0815 mmol) were added. The mixture was stirred for 6 h at room temperature. The solution was concentrated and purified by column chromatography on silica gel (petroleum ether/EtOAc, 2:1) to give **16** (30 mg, 77%). ^1^H NMR (600 MHz, CDCl_3_): *δ* 8.10-7.38 (m, 15H, ArH), 5.47 (t, *J* =9.2 Hz, 1H, H-3), 5.43 (dd, *J* = 9.8, 7.7 Hz, 1H, H-2), 4.79 (dd, *J* = 12.1, 4.4 Hz, 1H, H-6a), 4.73 (d, *J* = 7.6 Hz, 1H, H-1), 4.67 (dd, *J* = 12.1, 2.3 Hz, 1H, H-6b), 4.00 – 3.96 (m, 1H, H-α), 3.90 (td, 1H, *J* = 9.5, 4.2 Hz, H-4), 3.81 (ddd, *J* = 9.7, 4.3, 2.3 Hz, 1H, H-5), 3.61 (ddd, *J* = 9.9, 7.9, 4.7 Hz, 1H, H-α), 3.46 (d, *J* = 4.3 Hz, 1H, O*H*), 3.27 – 3.18 (m, 2H, H-γ), 1.87 – 1.70 (m, 2H, H-β).

*3-Azidopropyl(2,3,4,6-tetra-O-acetyl-β-D-galactopyranosyl)-(1→3)-(4,6-di-O-acetyl-2-acetamido-2-deoxy-β-D-galactopyranosyl)-(1→4)-(2,6-di-O-acetyl-3-O-tert- butyldimethysilyl-β-D-galactopyranoside**)-(1→4)-2,3,6-tri-O-benzoyl-β-D –glucopyranoside* (**18**) *–* A suspension of compound **17** (Zhang et al. 2015) (206 mg, 180 µmol), **16** (135 mg, 234 μmol) and molecular sieves (AW-300, 200 mg) in CH_2_Cl_2_ (5.0 ml) was stirred for 0.5 h, and then cooled to 0ºC. To the mixture was added TMSOTf (19 μl, 35 μmol) and stirring was continued for 1 h. Completion of the reaction was confirmed by TLC (petroleum ether/EtOAc, 2:3). Et_3_N was added to quench the reaction. The mixture was filtered through Celite. The filtrate was washed with satd NaHCO_3_ (2×20 ml), and brine (2×20 ml), dried over Na_2_SO_4_, and concentrated. The residue was purified by column chromatography on silica gel (petroleum ether/EtOAc, 1:1) to give **18** (238 mg, 85%) as a white solid. ^1^H NMR (600 MHz, CDCl_3_) *δ* 8.08-7.36(m, 15H, Ar-H), 6.64 (d, *J* = 6.3 Hz, 1H, -NH), 5.64 (t, *J* = 9.5 Hz, 1H, H-3), 5.43 (dd, *J* = 9.9, 7.9 Hz, 1H, H-2), 5.33 (d, *J* = 2.8 Hz, 1H, H-4ʹʹʹ), 5.28 (d, *J* = 3.7 Hz, 1H, H-4ʹʹ), 5.24 (d, *J* = 8.2 Hz, 1H, H-1ʹʹ), 5.13 (dd, *J* = 10.4, 7.9 Hz, 1H, H-2ʹʹʹ), 5.07 (dd, *J* = 11.0, 3.7 Hz, 1H, H-3ʹʹ), 5.02 (dd, *J* = 9.8, 7.7 Hz, 1H, H-2ʹ), 4.93 (dd, *J* = 10.5, 3.5 Hz, 1H, H-3ʹʹʹ), 4.77 (dd, *J* = 11.9, 2.0 Hz, 1H, H-6a), 4.69 (d, *J* = 7.9 Hz, 1H, H-1), 4.58 (d, *J* = 7.9 Hz, 1H, H-1ʹʹʹ), 4.39 (dd, *J* = 11.9, 5.4 Hz, 1H, H-6b), 4.36 (d, *J* = 7.7 Hz, 1H, H-1ʹ), 4.10 – 4.05 (m, 4H, H-4, H-6aʹʹ, H-6ʹʹʹ), 3.95 – 3.91 (m, 1H, H-α), 3.89 (ddd, *J* = 9.5, 5.3 1.8 Hz, 1H, H-5), 3.85 - 3.83 (m, 2H, H-5ʹʹʹ, H-4ʹ), 3.77 (dd, *J* = 11.6, 7.5 Hz, 1H, H-6bʹʹ), 3.71 (dd, *J* = 11.5, 5.3 Hz, 1H, H-6aʹ), 3.63 (dd, *J* = 7.7, 4.3 Hz, 1H, H-5ʹʹ), 3.60 – 3.56 (m, 1H, H-α), 3.54 (dd, *J* = 9.8, 2.4 Hz, 1H, H-3ʹ), 3.35 (t, *J* = 6.1 Hz, 1H, H-5ʹ), 3.23 – 3.16 (m, 3H, H-γ, H-6bʹ), 2.63 – 2.58 (m, 1H, H-2ʹʹ), 2.15, 2.14, 2.12, 2.07, 2.04, 2.02, 2.00 , 1.98, 1.95 (s, 27H, 9×C*H*_3_CO), 1.81 – 1.67 (m, 2H, H-β), 0.83 (s, 9H, *t*Bu), 0.01 (s, 3H, SiC*H*_3_), -0.01 (s, 3H, SiC*H*_3_). ^13^C NMR (150 MHz, CDCl­_3_) *δ* 172.68, 172.11, 170.48, 170.46, 170.33, 170.14, 169.96, 169.62, 169.43, 166.37, 166.13, 165.26, 133.61, 133.51, 133.48, 129.84, 129.83, 129.78, 129.74, 129.68, 129.51, 129.24, 128.74, 128.54, 101.43, 101.32, 100.13, 96.90, 74.94, 73.92, 73.65, 73.45, 73.28, 72.54, 72.06, 71.63, 71.10, 70.92, 70.88, 70.45, 69.53, 69.00, 67.02, 66.72, 63.11, 62.91, 62.77, 61.16, 56.15, 47.91, 29.03, 25.74, 23.72, 21.12, 20.94, 20.88, 20.86, 20.79, 20.76, 20.65, 18.03, -4.58, -4.96. HR ESI-MS: *m/z* calculated for C_72_H_92_O_32_N_4_SiNa [M+Na]^+^ 1575.5356, found 1575.5377.

*3-Azidopropyl (2,3,4,6-tetra-O-acetyl-β-D-galactopyranosyl)-(1→3)-(4,6-di-O- acetyl-2-acetamido-2-deoxy-β-D-galactopyranosyl)-(1→4)-(2,6-di-O-acetyl-β-D-galactopyranoside)-(1→4)-2,3,6-tri-O-benzoyl-β-D–glucopyranoside* (**19**) *–* AcOH and TBAF (1:1, 1.0 M solution in THF, 450 μl, 450 μmol) were added to a solution of **18** (140 mg, 90 μmol) in THF (1.5 ml) at 0°C. The mixture was stirred at rt for 3 h (completion of the reaction was confirmed by TLC, EtOAc/toluene, 1:1). The reaction mixture was diluted with CHCl_3_ (30 mL), and the solution was washed with saturated aqueous NaHCO_3_ (10 ml) and brine (10 ml), dried (Na_2_SO_4_), and concentrated. The residue was purified by column chromatography on silica gel (EtOAc/toluene, 1:2) to give a white solid **19** (119 mg, 92%). ^1^H NMR (500 MHz, CDCl_3_) *δ* 8.11 – 7.38 (m, 15H, Ar-H), 5.97 (d, *J* = 6.2 Hz, 1H, N*H*), 5.69 (t, *J* = 9.5 Hz, 1H, H-3), 5.42 (d, *J* = 8.1 Hz, 1H, H-1ʹʹ), 5.38 (d,d *J* =9.7, 8.0 Hz, 1H, H-2), 5.36(d, *J* = 3.5 Hz, 1H, H-4ʹʹ), 5.34 (d, *J* = 3.3 Hz, 1H, H-4ʹʹʹ), 5.14 (dd, *J* = 10.4, 7.8 Hz, 1H, H-2ʹʹʹ), 4.96 (dd, *J* = 10.4, 3.4 Hz, 1H, H-3ʹʹʹ), 4.81 (dd, *J* = 11.9, 1.8 Hz, 1H, H-6a), 4.76 (dd, *J* = 10.1, 7.8 Hz, 1H, H-2ʹ), 4.71 (d, *J* = 7.9 Hz, 1H, H-1), 4.55 (d, *J* = 7.8 Hz, 1H, H-1ʹʹʹ), 4.50 – 4.45 (m, 2H, H-6b, H-1ʹ), 4.37 (dd, *J* = 11.0, 3.5 Hz, 1H, H-3ʹʹ), 4.18 – 4.03 (m, 4H, H-4, H-6ʹʹʹ, H-6aʹʹ), 3.96 – 3.80 (m, 6H, H-5, H-5ʹʹʹ, H-α, H-4ʹ, H-6aʹ, H-6bʹʹ), 3.73 (t, *J* = 6.2 Hz, 1H, H-5ʹʹ), 3.58 (ddd, *J* = 9.9, 7.7, 4.9 Hz, 1H, H-α), 3.49 (dd, *J* = 11.5, 6.2 Hz, 1H, H-6bʹ), 3.43 (d, *J* = 9.7 Hz, 1H, H-3ʹ), 3.31 (t, *J* = 5.9 Hz, 1H, H-5ʹ), 3.24 – 3.16 (m, 3H, H-γ, OH), 2.96 – 2.89 (m, 1H, H-2ʹʹ), 2.15, 2.14, 2.07, 2.06, 2.04, 2.03, 2.02, 1.99, 1.97 (s, 27H, 9×COC*H*_3_), 1.85 – 1.65 (m, 2H, H-β). ^13^C NMR (125 MHz, CDCl_3_) *δ* 173.04, 170.81, 170.63, 170.49, 170.34, 170.31, 170.14, 169.78, 169.46, 166.18, 165.78, 165.36, 133.58, 133.47, 133.33, 129.93, 129.87, 129.79, 129.36, 128.77, 128.58, 128.50, 101.35, 100.50, 100.12, 98.79, 75.46, 75.23, 73.93, 73.41, 73.12, 73.01, 72.92, 72.35, 72.12, 71.67, 71.02, 69.47, 68.56, 66.95, 66.75, 62.89, 62.82, 62.43, 61.13, 55.89, 48.01, 29.12, 23.66, 21.03, 20.96, 20.93, 20.91, 20.87, 20.82, 20.80, 20.67. HR ESI-MS: *m/z* calculated for C_66_H_78_O_32_N_4_Na [M+Na]^+^ 1461.4419; found 1461.4523.

*3-Azidopropyl(2,3,4,6-tetra-O-acetyl-β-D-galactopyranosyl)-(1→3)-(4,6-di-O-acetyl-2-acetamido-2-deoxy-β-D-galactopyranosyl)-(1→4)-(2,6-di-O-acetyl-3-O-sulfonate-β-D-galactopyranoside)-(1→4)-2,3,6-tri-O-benzoyl-β-D–glucopyranoside* (**20**) *–* SO_3_⋅Py (80 mg, 502 μmol) was added to a solution of **19** (80 mg, 55 μmol) in DMF (1.5 ml). The mixture was stirred for 36 h at 90°C (completion of the reaction was confirmed by TLC, CH_2_Cl_2_/MeOH 17:1). The reaction mixture was concentrated, and the residue was diluted with CHCl_3_ (30 ml), and the solution was washed with saturated brine (10 ml), dried (Na_2_SO_4_), and concentrated. The residue was purified by column chromatography on silica gel (CH_2_Cl_2_/MeOH, 25:1) to give a white solid **20** (80 mg, 95%). ^1^H NMR (500 MHz, CDCl_3_) *δ* 8.09 – 7.36 (m, 15H, ArH), 5.64 (m, 1H, H-3), 5.40 (dd, *J* = 9.8, 8.1 Hz, 1H, H-2), 5.32 (m, 2.5H, H-4ʹʹ, H-4ʹʹʹ, H-1ʹʹ), 4.99 (m, 2H, H-3ʹʹʹ, H-2ʹʹʹ), 4.87 (m, 2H, H-6a, H-2ʹ), 4.72 (m, 2H, H-1, H-1ʹʹʹ), 4.59 (d, *J* = 7.3 Hz, 1H, H-1ʹ), 4.49 (d, *J* = 8.4 Hz, 0.5H, H-1ʹʹ), 4.36 (m, 4H, H-6b, H-6ʹʹ, H-3ʹ), 3.58 (ddd, *J* = 9.9, 7.8, 4.9 Hz, 1H, H-α), 3.21 (m, 2H, H-γ), 2.17 – 1.90 (m, 27H, 9×COC*H*_3_), 1.75 (m, 2H, H-β). ^13^C NMR (126 MHz, CDCl_3_) δ 172.82, 171.32, 170.68, 170.24, 169.90, 169.35, 165.90, 165.21, 165.14, 133.44, 129.69, 129.65, 128.60, 128.43, 128.27, 101.18, 101.01, 100.06, 99.50, 73.31, 73.10, 71.90, 71.72, 70.79, 70.10, 69.33, 67.07, 66.58, 62.59, 62.47, 60.89, 47.83, 28.94, 21.03, 20.96, 20.85, 20.76, 20.68, 20.53. HR ESI-MS: *m/z* calculated for C_66_H_78_O_35_N_4_SNa [M+Na]^+^ 1541.4060, found 1541.4104.

*3-Azidopropyl-β-D-galactopyranosyl-(1→3)-2-acetamido-2-deoxy-β-D-galactopyranosyl-(1→4)-(3-O-sulfonate-β-D-galactopyranoside)-(1→4)-β-D-glucop­y­rano­s­ide* (**21**) *–* A catalytic amount of sodium methoxide (25% in MeOH) was added to a solution of **20** (40.0 mg, 26 μmol) in a mixture of MeOH (400 μl) and THF (300 μl) at rt. The mixture was stirred for 5h at rt and completion of the reaction was confirmed by TLC (CHCl_3_/MeOH/H_2_O 5:5:1). The reaction mixture was neutralized with Dowex-50 (H^+^). The mixture was filtered through cotton and washed with mixed solvent (CHCl_3_/MeOH, 1:1). The combined filtrate was concentrated. The residue was purified by column chromatography on silica gel (CHCl_3_/MeOH/H_2_O, 5:5:1), then column-chromatography (CH_3_OH) on Sephadex LH-20 to give **21** (22 mg, 95%). ^1^H NMR (500 MHz, CD_3_OD) *δ* 4.71 (d, *J* = 8.5 Hz, 1H, H-1ʹʹ), 4.46 (d, *J* = 7.7 Hz, 1H, H-1ʹ), 4.38 (d, *J* = 6.9 Hz, 1H, H-1ʹʹʹ), 4.32 (d, *J* = 9.9 Hz, 1H, H-3ʹ), 4.29 (d, *J* = 7.8 Hz, 1H, H-1), 4.12 (t, *J* = 9.5 Hz, 1H, H-2ʹʹ), 4.06 (s, 1H, H-4ʹʹ), 3.98 – 3.91 (m, 1H, H-α), 3.91 – 3.83 (m, 4H, H-3ʹʹʹ, H-4, H-6^x^), 3.82 – 3.60 (m, 8H, H-3ʹʹ, H-6^x^, H-6^x^, H-6^x^, H-α), 3.60 – 3.46 (m, 7H, H-5ʹʹʹ, H-5ʹ, H-5ʹʹ, H-2ʹʹʹ, H-2ʹ, H-3, H-4ʹʹʹ), 3.44 (t, *J* = 6.7 Hz, 2H, H-γ), 3.39 (d, *J* = 9.3 Hz, 1H, H-5), 3.30 (s, 1H, H-4ʹ), 3.26 (t, *J* = 8.4 Hz, 1H, H-2), 2.06 (s, 3H, COC*H*_3_), 1.90 – 1.83 (m, 2H, H-β). ^13^C NMR (125 MHz, CD_3_OD) *δ* 173.70, 105.14, 103.38, 102.81, 80.42, 79.55, 79.51, 78.02, 77.76, 77.50, 75.20, 74.96, 74.75, 74.71, 74.10, 73.29, 73.08, 71.05, 69.43, 68.90, 68.10, 66.36, 61.23, 60.39, 59.97, 51.47, 28.85, 22.33. HR ESI-MS: *m/z* calculated for C_29_H_49_O_24_N_4_S [M-H]^-^ 869.2452, found 869.2453.

**21a**: ^1^H NMR (500 MHz, CD_3_OD) *δ* 4.72 (d, *J* = 8.1 Hz, 1H, H-1ʹʹ), 4.52 (d, *J* = 7.6 Hz, 1H, H-1ʹ), 4.38 (d, *J* = 7.0 Hz, 2H, H-1ʹʹʹ), 4.29 (d, *J* = 7.9 Hz, 1H, H-1), 4.25 (d, *J* = 8.4 Hz, 1H, H-2ʹ), 4.11 – 4.04 (m, 2H, H-2ʹʹ, H-4ʹʹ) , 3.99 – 3.82 (m, 5H, H-α, H-3ʹʹʹ, H-4, H-6^x^), 3.82 – 3.60 (m, 8H, H-3ʹʹ, H-6^x^, H-6^x^, H-6^x^, H-α), 3.59 – 3.48 (m, 7H, H-5ʹʹʹ, H-5ʹ, H-5ʹʹ, H-2ʹʹʹ, H-3ʹ, H-3, H-4ʹʹʹ), 3.47 – 3.39 (m, 3H, H-γ, H-5), 3.32 – 3.28 (m, 1H, H-4ʹ), 3.25 (t, *J* = 8.3 Hz, 1H), 2.11 (s, 3H, COC*H*_3_), 1.91 – 1.83 (m, 2H, H-β). ^13^C NMR (125 MHz, CD_3_OD): *δ* 174.08, 105.00, 102.73, 102.60, 101.30, 79.55, 78.26, 75.12, 75.02, 74.81, 74.70, 74.45, 73.35, 73.28, 73.11, 73.13, 66.33, 61.34, 61.25, 60.22, 28.85, 21.79. HR ESI-MS: *m/z* calculated for C_29_H_49_O_24_N_4_S [M-H]^-^ 869.2452, found 869.2453.

*3-Aminoethyl-β-D-galactopyranosyl-(1→3)-2-acetamido-2-deoxy-β-D-galactopyranosyl-(1→4)-(3-O-sulfonate-β-D-galactopyranoside)-(1→4)-β-D-glucop­y­rano­s­ide* (**22**, **SM1a-C3-NH_2_**) *–* 10% Pd/C (5 mg) was added to a solution of compound **21** in methanol (1 mL). The mixture was stirred at room temperature under a hydrogen atmosphere (balloon) for 48 h. The mixture was filtered through Celite, and the filtrate was concentrated in vacuo to give compound **SM1a-C3-NH_2_ (22)** (1.7 mg, 87%). HR ESI-MS: *m/z* calculated for C_29_H_51_N_2_O_24_S [M−H]^−^ 843.2552, found 843.2402.

*3-Aminoethyl-β-D-galactropyranosyl-(1→3)-2-acetamido-2-deoxy-β-D-galactopy-ranosyl-(1→4)-(2-O-sulfonate-β-D-galactopyranoside)-(1→4)-β-D-glucopyrano- side* (**23**, **SM1a(2S)-C3-NH_2_**) *–*10% Pd/C (5 mg) was added to a solution of compound **21a** in methanol (1 mL). The mixture was stirred at room temperature under a hydrogen atmosphere (balloon) for 48 h. The mixture was filtered through Celite, and the filtrate was concentrated in vacuo to give compound **SM1a(2S)-C3-NH_2_ (23)** (1.9 mg, 99%). HR ESI-MS: *m/z* calculated for C_29_H_51_N_2_O_24_S [M−H]^−^ 843.2552; found 843.2655.

**Synthesis of methylamino-terminating glycans**

Using 2-(methylamino)ethyl galactoside (**Gal-C2-NHMe, 27**) as an example, the preparation of methylamino-terminating glycans can be described as follows. **Glc-C2-NHMe (28)** and **Lac-C2-NHMe (29)** were similarly prepared.

**Supplementary Scheme 4**. Synthesis of Gal-C2-NHMe (**27**), Glc-C2-NHMe (**28**) and Lac-C2-NHMe (**29**): a) 2-[(N-benzyloxycarbonyl) methylamino] ethanol, TMSOTf, DCM, -15 ºC, 70%; b) e) 10% Pd/C, H_2_, 40 ºC, 5 h, 96%; c) CH_3_ONa/CH_3_OH, pH 8-9, 67%.

*2-[(N-benzyloxycarbonyl)methylamino]ethyl-2,3,4,6-Tetra-O-benzoyl-β-D-galactopyranoside* (**25**) *−* A mixture of 2,3,4,6-tetra-*O*-benzoyl-D-galactopyranosyl trichloroacetimidate **24** (Lee et al. 2011) (828 mg, 1.12 mmol) and 2-[(N-benzyloxycarbonyl) methylamino] ethanol (Mohler and Shen, 2006) (352 mg, 1.7 mmol) in dry 15mL CH_2_Cl_2_ with 4 Å molcular sieve was stirred under nitrogen atmosphere, cooled to -15 °C, and TMSOTf (30 μL, 0.17 mmol) was added. After stirring for 1 h at -15^o^C, the reaction was quenched with the addition of Et_3_N. Subsequently, the mixture was filtered and the filtrate was concentrated under reduced pressure. The crude product was purified by column chromatography (EtOAc/petroleum ether, 1:6, v/v) to give **25** (617 mg, 70%). ^1^H NMR (400 MHz, CDCl_3_) *δ* 8.13 (d, *J* = 7.2 Hz, 2H, ArH), 8.06 (d, *J* = 7.3 Hz, 2H, ArH), 7.98 (d, *J* = 7.3 Hz, 2H, ArH), 7.82 (d, J = 7.3 Hz, 2H, ArH), 7.70 – 7.24 (m, 17H, ArH), 6.04 (s, 1H, H-4), 5.84 (q, *J* = 10.4 Hz, 1H, H-2), 5.70 – 5.57 (m, 1H, H-3), 5.18 – 4.95 (m, 2H, -OC***H_2_***Ph), 4.89 (d, *J* = 7.9 Hz, 0.6H, H-1), 4.79 (d, *J* = 7.8 Hz, 0.5H, H-1), 4.70 (dd, *J* = 11.2, 6.7 Hz, 1H, H-6_a_), 4.52 – 4.40 (m, 1H, H-6_b_), 4.37 (t, *J* = 6.3 Hz, 0.6H, H-5), 4.27 (t, *J* = 6.5 Hz, 0.5H, H-5), 4.21 – 4.03 (m, 1H, -OC***Ha***H_b_CH_2_NCH_3_-), 3.92 – 3.80 (m, 0.6H, -OCHa***H_b_***CH_2_NCH_3_-), 3.79 – 3.68 (m, 0.5H, -OCHa***H_b_***CH_2_NCH_3_-), 3.67 – 3.51 (m, 1H, -OCH_2_C***Ha***H_b_NCH_3_-), 3.49 – 3.36 (m, 1H, -OCH_2_CHa***H_b_***NCH_3_-), 2.88 (s, 1.8H, -OCH_2_CH_2_NC***H_3_***-), 2.86 (s, 1.5H, -OCH_2_CH_2_NC***H_3_***-). HR ESI-MS: *m/z* calculated for C_45_H_41_NO_12_Na [M+Na]^+^ 810.2526, found 810.2519.

*2-(methylamino)ethyl galactoside* (**27**) (Pettenuzzo et al. 2019) *−* **25** (200 mg, 0.3 mmol) was dissolved in 6mL THF and 10% Pd/C (100 mg) was added. The reaction was stirred under a hydrogen atmosphere at 40℃ for 5 h. The solution was then filtered through celite and the solvent removed under reduced pressure. The residue was purified by column chromatography on silica (methylene dichloride-methanol =30:1, V/V) to afford pure **26** (159 mg, 96%). ^1^H NMR (500 MHz, CDCl_3_) *δ* 8.09 (d, *J* = 8.1 Hz, 2H, ArH), 8.02 (d, *J* = 8.2 Hz, 2H, ArH), 7.97 (d, *J* = 7.8 Hz, 2H, ArH), 7.79 (d, *J* = 8.2 Hz, 2H, ArH), 7.68 – 7.32 (m, 9H, ArH), 7.24 (t, *J* = 7.7 Hz, 2H, ArH), 6.01 (d, *J* = 3.2 Hz, 1H, H-4), 5.79 (dd, *J* = 10.1, 8.2 Hz, 1H, H-2), 5.63 (dd, *J* = 10.4, 3.3 Hz, 1H, H-3), 4.87 (d, *J* = 7.9 Hz, 1H, H-1), 4.68 (dd, *J* = 11.3, 6.4 Hz, 1H, H-6_a_), 4.44 (dd, *J* = 11.3, 6.6 Hz, 1H, H-6_b_), 4.35 (t, *J* = 6.6 Hz, 1H, H-5), 4.14 – 4.04 (m, 1H, -OC***H_a_***H_b_CH_2_NHCH_3_), 3.80 – 3.69 (m, 1H, -OCH_a_***H_b_***CH_2_NHCH_3_), 2.86 – 2.75 (m, 1H, -OCH_2_C***H_a_***H_b_NHCH_3_), 2.74 – 2.63 (m, 1H, -OCH_2_CH_a_***H_b_***NHCH_3_), 2.24 (s, 3H, -OCH_2_CH_2_NHC***H_3_***). ^13^C NMR (126 MHz, CDCl_3_) δ 166.17, 165.66, 165.43, 133.95 – 133.07 (m), 130.74 – 127.89 (m), 102.10, 71.76, 71.49, 70.00, 69.90, 68.24, 62.16, 51.03, 36.05. ESI-MS: *m/z* calculated for C_37_H_36_NO_10_[M+H]^+^ 654.2, found 654.3.

Compound **26** (29 mg, 0.044 mmol) was dissolved in 2 ml of methanol, and then sodium methoxide was added to the reaction mixture until pH value was 8~9. Then the reaction mixture was stirred at room temperature for 1 h. Subsequently, ion-exchange resin (Amberlite IR 120 H form, strongly acidic) was added and pH of the reaction mixture was adjusted to 7. The reaction mixture was filtered and the filtrate was evaporated to give **27** as a white solid (7 mg, 67%). ^1^H NMR (400 MHz, CD_3_OD) *δ* 4.22 (d, *J* = 7.3 Hz, 1H, H-1), 4.03 – 3.94 (m, 1H, -OC***H_a_***H_b_CH_2_NHCH_3_), 3.82 (d, *J* = 3.0 Hz, 1H, H-4), 3.80 – 3.66 (m, 3H, H-6_a_, H-6_b_, -OCH_a_***H_b_***CH_2_NHCH_3_), 3.58 – 3.43 (m, 3H, H-2, H-3, H-5), 2.78 (t, *J* = 5.1 Hz, 2H, -OCH_2_C***H_2_***NHCH_3_), 2.41(s, 3H, -OCH_2_CH_2_NHC***H_3_***). ^13^C NMR (126 MHz, CD_3_OD) δ 103.70, 75.35, 73.40, 71.19, 68.89, 67.46, 61.13, 50.39, 34.21. ESI-MS: *m/z* calculated for C_9_H_20_NO_6_ [M+H]^+^ 238.1, found 238.3.

Glc-C2-NHMe (Pettenuzzo et al. 2019) (**28**) and Lac-C2-NHMe (**29**) were similarly prepared with the following analytical data.

Glc-C2-NHMe (**28**): ^1^H NMR (400 MHz, CD_3_OD) *δ* 4.24 (d, *J* = 7.7 Hz, 1H, H-1), 3.98 (dt, *J* = 9.5, 4.3 Hz, 1H, -OC***H_a_***H_b_CH_2_NHCH_3_), 3.84 (d, *J* = 11.8 Hz, 1H, H-6_a_), 3.74 – 3.57 (m, 2H, -OCH_a_***H_b_***CH_2_NHCH_3_, H-6_b_), 3.39 – 3.31 (m, 1H, H-3), 3.28 – 3.22 (m, 1H, H-4, H-5), 3.18 (t, *J* = 8.4 Hz, 1H, H-2), 2.81 – 2.68 (m, -OCH_2_C***H_2_***NHCH_3_), 2.39 (s, 3H, -OCH_2_CH_2_NHC***H_3_***). ^13^C NMR (126 MHz, CD_3_OD) δ 103.10, 76.58, 76.46, 73.72, 70.21, 67.67, 61.33, 50.40, 34.33. HR ESI-MS: *m/z* calculated for C_9_H_20_NO_6_ [M+H]^+^ 238.1291, found 238.1285.

Lac-C2-NHMe (**29**): ^1^H NMR (500 MHz, D_2_O) *δ* 4.56 (d, *J* = 7.9 Hz, 1H, H-1), 4.47 (d, *J* = 7.8 Hz, 1H, H-1'), 4.18 (dd, *J* = 11.9, 4.9 Hz, 1H, -OC***H_a_***H_b_CH_2_NHCH_3_), 4.00 (dd, *J* = 11.4, 5.7 Hz, 2H, -OCH_a_***H_b_***CH_2_NHCH_3_, H-6_a_), 3.95 (d, *J* = 3.3 Hz, 1H, H-4'), 3.88 – 3.72 (m, 4H, H-6_b_, H-5', H-6_a_', H-6_b_'), 3.72 – 3.61 (m, 4H, H-3, H-4, H-5, H-3'), 3.60 – 3.53 (m, 1H, H-2'), 3.39 (dd, *J* = 15.4, 6.4 Hz, 1H, H-2), 3.33 (t, *J* = 4.8 Hz, 2H, -OCH_2_C***H_2_***NHCH_3_), 2.78 (s, 3H, -OCH_2_CH_2_NHC***H_3_***). HR ESI-MS: *m/z* calculated for C_15_H_30_NO_11_ [M+H]^+^ 400.1819, found 400.1885.

**Synthesis of GalNAc and GlcNAc linked to Ser and Thr**

**Supplementary Scheme 5.** Synthesis of GalNAc- and GlcNAc-Ser/Thr amino acids; i: Fmoc-Thr-*O-t*Bu or Fmoc-Ser-*O-t*Bu, Ag_2_CO_3_, AgClO_4_, MS4Å, DCM/Tol (1:1), r.t., 24 h; ii: Zn, AcOH, r.t., 24 h; iii: Ac_2_O, Pyr, r.t., 16 h; iv: TFA/DCM (1:3), r.t., 3 h, v: NaOMe, MeOH, pH 11, 48 h.

*N-9-Fluorenylmethoxycarbonyl-O-[3,4,6-tri-O-acetyl-2-deoxy-2-N-(2,2,2-trichloroethoxycarbonylamino)-β-D-galactopyranosyl]-L-threonine-tert-butylester* (**31**) *−* FmocThrOtBu (Zhu and Boons, 1999) (3.84 g, 9.68 mmol, 1.0 eq) was dissolved in anhydrous DCM/Toluene 1:1 solvent (76 mL) with preheated 4 Å molecular sieves (7.61 g), stirred at r.t. for 30 min. Silver perchlorate monohydrate (872 mg, 3.87 mmol, 0.4 eq) (pre coevaporated with toluene (10 mL×3)) and silver carbonate (5.34 g, 19.4 mmol, 2.0 eq) were added into the mixture, kept stirring for another 30 min in darkness. A solution of **30** (Kunz, 1997) (7.36 g, 13.5 mmol, 1.4 eq) in anhydrous DCM/Toluene 1:1 (110 mL) was added slowly over 1 h, and the reaction was stirred at r.t. for 24 h followed by TLC. The reaction mixture was diluted with DCM (300 mL), washed with Sat. NaHCO_3_ (200 mL×3), H_2_O (200 mL×2), brine (300 mL×1), dried over MgSO_4_, concentrated and purified by column chromatography (cyclohexane-ethyl acetate 4:1 to 3:1) to give **32** yield 93% (7.73 g, 8.99 mmol). R*_f_* = 0.4 (^C^Hex/EtOAc 2:1). *HR-ESI-MS (pos), m/z*: 859.2019, 861.1999 ([M+H]^+^, calculated 859.2015, 861.1985), 881.1849, 883.1821 ([M+Na]^+^, calculated 881.1834, 883.1805).

***^1^H-NMR*** (400 MHz, CDCl_3,_ gCOSY, gHSQC, gHMBC), *δ (ppm)*: 7.75 (dd, *J* = 7.5, 1.1 Hz, 2H, Fmoc), 7.66 (dd, *J* = 7.7, 2.8 Hz, 2H), 7.38 (td, *J* = 7.4, 1.2 Hz, 2H, Fmoc)), 7.31 (tt, *J* = 7.5, 1.6 Hz, 2H, Fmoc), 5.71 (d, *J* = 9.4 Hz, 1H, ThrN*H* ), 5.35 (d, *J* = 3.1 Hz, 1H, H4), 5.20 (dd, *J* = 11.6, 3.1 Hz, 1H, H3), 4.79 – 4.60 (m, 3H, TrocC*H*_2_, H1), 4.54 – 4.39 (m, 2H, FmocC*H*_2_, Thr-C*H*β), 4.31 – 4.22 (m, 3H, FmocC*H*_2_, H6a, FmocC*H*, Thr-C*H*α), 4.17 – 4.04 (m, 2H, H6ab), 3.89 (t, *J* = 6.7 Hz, 1H, H5), 3.75 (td, *J* = 9.5, 5.2 Hz, 1H, H2), 2.10 (s, 3H), 2.04 (s, 3H), 2.00 (s, 3H) (2.10 – 2.00 Ac), 1.49 (s, 9H, tBu), 1.20 (d, *J* = 6.2 Hz, 3H, Thr-C*H*_3_).

***^13^C-NMR*** (100.6 MHz, CDCl_3_, gHSQC, gHMBC), *δ (ppm)*: 170.76, 170.63, 170.30, 169.19, 157.06, 154.54, 144.16, 144.08, 141.49, 141.45, 127.92, 127.35, 127.32, 125.53, 120.16 (127.92 – 120.16 Fmoc), 97.81 (C1), 95.63 (*C*Cl_3_), 82.33 (*C*(CH_3_)_3_), 74.72 (TrocCH_2_), 73.10 (Thr-*C*Hβ), 70.75 (C5), 69.65 (C3), 67.53 (Fmoc*C*H_2_), 66.81 (C4), 61.61 (C6), 59.16 (Thr-*C*Hα), 53.14 (C2), 47.41 (Fmoc*C*H), 28.11 (tBu), 27.12, 20.89, 20.87, 20.77 (20.89 – 20.77 Ac), 16.05 (Thr-*C*H_3_).

*N-9-Fluorenylmethoxycarbonyl-O-(2-N-acetamido-3,4,6-tri-O-acetyl-2-deoxy-β-D-galactopyranosyl)-L-threonine-tert-butylester* (**32**) *−* Zinc powder was activated by treatment with 1 M HCl aq. for 20 min, washed with H_2_O, MeOH, Et_2_O and dried under reduced pressure for 30 min. Compound **31** (7.7 g, 8.95 mmol, 1.0 eq) was dissolved in glacial acetic acid (150 mL) followed by addition of the activated Zn powder (9.31 g, 143.2 mmol, 16 eq). The reaction mixture was stirred at room temperature for 24 h and was then filtered through a thin layer of *Celite*. The filtrate was concentrated and coevaporated 3 times with toluene. The residue was dissolved in pyridine / acetic anhydride 2:1 (60 mL) and stirred at room temperature overnight. Then the reaction mixture was concentrated and coevaporated three times with toluene. Purification by column chromatography (cyclohexane-ethyl acetate 4:1 to 1:2) gave **32**. Yield: 92 % (5.98 g, 8.23 mmol). R*_f_* = 0.4 (^C^Hex/EtOAc 1:2). *HR-ESI-MS (pos), m/z*: 727.3068 ([M+H]^+^, calculated 727.3033), 749.2887 ([M+Na]^+^, calculated 749.2898).

***^1^H-NMR*** (400 MHz, CDCl_3,_ gCOSY, gHSQC, gHMBC), *δ (ppm)*: 7.76 (d, *J* = 7.5 Hz, 2H, Fmoc), 7.71 – 7.65 (m, 2H, Fmoc), 7.39 (t, *J* = 7.4 Hz, 2H, Fmoc), 7.32 (t, *J* = 7.6 Hz, 2H, Fmoc), 5.98 (d, *J* = 8.5 Hz, 1H, N*H*Ac), 5.78 (d, *J* = 9.1 Hz, 1H, ThrN*H*), 5.38 – 5.32 (m, 2H, H3, H4), 4.75 (d, *J* = 8.2 Hz, 1H, H1), 4.47 – 4.33 (m, 3H, FmocC*H*_2_, Thr-C*H*β), 4.29 – 4.21 (m, 2H, FmocC*H*, Thr-C*H*α), 4.16 – 4.08 (m, 2H, H6ab), 3.95 (t, *J* = 6.6 Hz, 1H, H5), 3.87 (dt, *J* = 11.2, 8.3 Hz, 1H, H2), 2.10 (s, 3H), 2.04 (s, 3H), 2.00 (s, 3H), 1.95 (s, 3H) (2.10 – 1.95 Ac), 1.50 (s, 9H, tBu), 1.18 (d, *J* = 6.3 Hz, 3H, Thr-C*H*_3_).

***^13^C-NMR*** (100.6 MHz, CDCl_3_, gHSQC, gHMBC), *δ (ppm)*: 171.21, 170.65, 170.55, 170.46, 170.21, 169.14, 156.89, 144.10, 143.85, 141.31, 127.72, 127.15, 127.11, 125.38, 125.31, 119.97 (127.72 – 119.97 Fmoc), 97.71 (C1), 82.12 (*C*(CH_3_)_3_), 73.27 (Thr-*C*Hβ), 70.52 (C5), 69.71 (C3), 67.10 (Fmoc*C*H_2_), 66.80 (C4), 61.56 (C6), 59.14 (Thr-*C*Hα), 51.82 (C2), 47.26 (Fmoc*C*H), 27.94 (tBu), 23.51, 20.76, 20.72, 20.63 (23.51 – 20.63 Ac), 16.08 (Thr-*C*H_3_).

*N-9-Fluorenylmethoxycarbonyl-O-(2-N-acetamido-3,4,6-tri-O-acetyl-2-deoxy-β-D-galactopyranosyl)-L-threonine* (**33**) *−* Compound **32** (5.92 g, 8.15 mmol) was dissolved in TFA/DCM 1:3 (120 mL), stirred at room temperature for 3 h. The solution was then concentrated and coevaporated with toluene (50 mL×3), concentrated and purification by silica column chromatography (DCM-MeOH 95:5 to 92:8) gave **33**. Yield: 90% (4.97 g, 7.31 mmol). R*_f_* = 0.3 (DCM/MeOH 9:1). *HR-ESI-MS (pos), m/z*: 671.2438 ([M+H]^+^ (calculated 671.2452).

***^1^H-NMR*** (400 MHz, CDCl_3,_ gCOSY, gHSQC, gHMBC), *δ (ppm)*: 7.74 (d, *J* = 7.5 Hz, 2H, Fmoc), 7.62 (dd, *J* = 7.4, 5.1 Hz, 2H, Fmoc), 7.37 (t, *J* = 7.4 Hz, 2H, Fmoc), 7.30 (dt, *J* = 8.6, 4.3 Hz, 2H, Fmoc), 6.63 (d, *J* = 8.8 Hz, 1H, N*H*Ac), 5.95 (d, *J* = 8.9 Hz, 1H, ThrN*H*), 5.32 (d, *J* = 3.4 Hz, 1H, H4), 5.21 (dd, *J* = 11.2, 3.4 Hz, 1H, H3), 4.72 (d, *J* = 8.4 Hz, 1H, H1), 4.51 – 4.35 (m, 4H, FmocC*H*_2_, Thr-C*H*β, Thr-C*H*α), 4.28 – 4.13 (m, 2H, FmocC*H*, H6a), 4.11 – 3.98 (m, 2H, H6b, H2), 3.90 (t, *J* = 6.7 Hz, 1H, H5), 2.11 (s, 3H), 1.99 (s, 3H), 1.97 (s, 6H) (2.11 – 1.97 Ac), 1.23 (d, *J* = 6.3 Hz, 3H, Thr-C*H*_3_).

***^13^C-NMR*** (100.6 MHz, CDCl_3_, gHSQC, gHMBC), *δ (ppm)*: 172.87, 171.85, 171.21, 170.97, 170.56, 157.16, 144.05, 143.91, 141.47, 127.98, 127.35, 125.42, 120.21 (127.98 – 120.21 Fmoc), 100.43 (C1), 75.77 (Thr-*C*Hβ), 70.78 (C5), 70.14 (C3), 67.55 (Fmoc*C*H_2_), 66.80 (C4), 61.51 (C6), 58.40 (Fmoc*C*H), 53.67 (Thr-*C*Hα), 51.57 (C2), 47.33(Fmoc*C*H), 23.30, 20.90 (23.30 – 20.90 Ac), 17.98 (Thr-*C*H_3_).

*(2-N-Acetamido-2-deoxy-β-D-galactopyranosyl)-L-threonine* (**34**) *−* Compound **33** (105 mg, 0.157 mmol) was dissolved in MeOH (10 mL), 1% NaOMe in MeOH was added to pH 11, stirred at room temperature for 48 h. After the reaction was finished, it was neutralized by 0.5 mL 1 M HCl, concentrated, and purification by silica column chromatography (DCM-MeOH-AcOH 10:1:1 to 1:1:1) gave **34**. Yield: 89% (45 mg, 0.139 mmol). R*_f_* = 0.3 (EtOAc/MeOH/H_2_O/AcOH 6:3:3:2). *HR-ESI-MS (pos), m/z*: 345.1260 ([M+Na]^+^, calculated 345.1274).

***^1^H-NMR*** (500 MHz, D_2_O_,_ gCOSY, gHSQC, gHMBC), *δ (ppm)*: 4.41 (d, *J* = 8.4 Hz, 1H, H1), 4.21 (p, *J* = 6.3 Hz, 1H, Thr-C*H*β), 3.86 (d, *J* = 3.2 Hz, 1H, H4), 3.82 (dd, *J* = 10.8, 8.3 Hz, 1H, H2), 3.76 (dd, *J* = 12.0, 8.3 Hz, 1H, H6a), 3.67 (t, *J* = 3.7 Hz, 1H, H6b), 3.65 (d, *J* = 3.0 Hz, 1H, H3), 3.59 (dd, *J* = 8.4, 3.7 Hz, 1H, H5), 3.55 (d, *J* = 5.8 Hz, 1H, Thr-C*H*α), 2.00 (s, 3H, Ac)1.24 (d, *J* = 6.4 Hz, 3H, Thr-C*H*_3_).

***^13^C-NMR*** (126 MHz, D_2_O, gHSQC, gHMBC), *δ (ppm)*: 175.00 (*C*OCH_3_), 172.23 (*C*OOH), 99.97 (C1), 75.08(C5), 73.99 (Thr-*C*Hβ), 70.65 (C3), 67.73 (C4), 61.19 (C6), 59.35 (Thr-*C*Hα), 52.22 (C2), 22.05 (Ac), 17.31 (Thr-*C*H_3_).

*N-9-Fluorenylmethoxycarbonyl-O-[3,4,6-tri-O-acetyl-2-deoxy-2-N-(2,2,2-trichloroethoxycarbonylamino)-β-D-galactopyranosyl]-L-serine-tert-butylester* (**35**) *−* FmocSerOtBu^[1]^ (1.0 g, 2.60 mmol, 1.0 eq) was dissolved in anhydrous DCM/Toluene 1:1 solvent (20 mL) with preheated 4 Å molecular sieves (2 g), stirred at r.t. for 30 min. Silver perchlorate monohydrate (235 mg, 1.04 mmol, 0.4 eq) (pre coevaporated with toluene (5 mL×3)) and silver carbonate (1.44 g, 5.20 mmol, 2.0 eq) were added into the mixture, kept stirring for another 30 min in darkness. A solution of **30** (Kunz, 1997) (1.85 g, 3.39 mmol, 1.4 eq) in anhydrous DCM/Toluene 1:1 (27.7 mL) was added slowly over 1 h, and the reaction was stirred at r.t. for 24 h followed by TLC. The reaction mixture was diluted with DCM (100 mL), washed with Sat. NaHCO_3_ (100 mL×3), H_2_O (100 mL×2), brine (100 mL×1), dried over MgSO_4_, concentrated and purified by column chromatography (cyclohexane-ethyl acetate 4:1 to 3:1) to give **35** yield 97% (2.13 g, 2.52 mmol). R*_f_* = 0.4 (^C^Hex/EtOAc 2:1). *HR-ESI-MS (pos), m/z*: 845.1864, 847.1842 ([M+H]^+^, calculated 845.1858, 847.1829), 867.1713, 869.1668 ([M+Na]^+^, calculated 867.1678, 869.1648).

***^1^H-NMR*** (400 MHz, CDCl_3,_ gCOSY, gHSQC, gHMBC), *δ (ppm)*: 7.75 (dt, *J* = 7.6, 0.9 Hz, 2H, Fmoc), 7.63 (dd, *J* = 11.0, 7.5 Hz, 2H, Fmoc), 7.39 (tdd, *J* = 7.5, 2.4, 1.2 Hz, 2H, Fmoc), 7.31 (tdd, *J* = 7.5, 5.0, 1.2 Hz, 2H, Fmoc), 5.80 (d, *J* = 8.3 Hz, 1H, SerN*H*), 5.35 (d, *J* = 2.3 Hz, 1H, H4), 5.13 (d, *J* = 11.6 Hz, 1H, H3), 4.82 – 4.72 (m, 1H, TrocC*H*_2_), 4.62 (d, *J* = 8.1 Hz, 1H, H1), 4.55 – 4.47 (m, 2H, TrocC*H*_2_, FmocC*H*_2_), 4.45 – 4.37 (m, 1H, Ser-C*H*α), 4.33 – 4.19 (m, 2H, FmocC*H*_2_, FmocC*H*), 4.19 – 4.05 (m, 3H, H6ab, Ser-C*H*_2_β), 3.93 – 3.83 (m, 2H, H5, Ser-C*H*_2_β), 3.79 (d, *J* = 8.8 Hz, 1H, H2), 2.11 (s, 3H), 2.02 (s, 3H), 1.97 (s, 3H) (2.11 – 1.97 Ac), 1.48 (s, 9H, tBu).

***^13^C-NMR*** (100.6 MHz, CDCl_3_, gHSQC, gHMBC), *δ (ppm)*: 170.60, 170.54, 170.32, 168.72, 156.38, 154.51, 144.21, 143.84, 141.53, 141.50, 127.99, 127.35, 125.49, 125.32, 120.24 (127.99 – 120.24 Fmoc), 100.70 (C1), 95.71 (*C*Cl_3_), 83.00 (*C*(CH_3_)_3_), 74.61 (TrocCH_2_), 70.95 (H5), 70.12 (H3), 69.30 (Ser-*C*H_2_β), 67.48 (Fmoc*C*H_2_), 66.88 (C4), 61.67 (C6), 54.67 (Ser-*C*Hα), 52.92 (C2), 47.32 (Fmoc*C*H), 28.13 (tBu), 20.85, 20.82, 20.80 (20.85 – 20.80 Ac).

*N-9-Fluorenylmethoxycarbonyl-O-(2-N-acetamido-3,4,6-tri-O-acetyl-2-deoxy-β-D-galactopyranosyl)-L-serine-tert-butylester* (**36**) *−* Zinc powder was activated by treatment with 1 M HCl aq. for 20 min, washed with H_2_O, MeOH, Et_2_O and dried under reduced pressure for 30 min. Compound **35** (2.1 g, 2.48 mmol, 1.0 eq) was dissolved in glacial acetic acid (40 mL) followed by addition of the activated Zn powder (2.58 g, 39.7 mmol, 16 eq). The reaction mixture was stirred at room temperature for 24 h and was then filtered through a thin layer of *Celite*. The filtrate was concentrated and coevaporated 3 times with toluene. The residue was dissolved in pyridine / acetic anhydride 2:1 (20 mL) and stirred at room temperature overnight. Then the reaction mixture was concentrated and coevaporated three times with toluene. Purification by column chromatography (cyclohexane-ethyl acetate 4:1 to 1:2) gave **36**. Yield: 88 % (1.56 g, 2.19 mmol). R*_f_* = 0.4 (^C^Hex/EtOAc 1:2). *HR-ESI-MS (pos), m/z*: 713.2908 ([M+H]^+^, calculated 713.2922), 735.2725 ([M+Na]^+^, calculated 735.2741).

***^1^H-NMR*** (400 MHz, CDCl_3,_ gCOSY, gHSQC, gHMBC), *δ (ppm)*: 7.76 (dd, *J* = 7.5, 3.2 Hz, 2H, Fmoc), 7.64 (d, *J* = 7.5 Hz, 2H, Fmoc), 7.43 – 7.34 (m, 2H, Fmoc), 7.31 (t, *J* = 7.4 Hz, 1H, Fmoc), 5.77 (d, *J* = 7.9 Hz, 1H, N*H*Ac), 5.67 (d, *J* = 8.6 Hz, 1H, SerN*H*), 5.33 (dd, *J* = 3.4, 1.1 Hz, 1H, H4), 5.22 (dd, *J* = 11.2, 3.4 Hz, 1H, H3), 4.66 (d, *J* = 8.4 Hz, 1H, H1), 4.51 – 4.28 (m, 3H, FmocC*H*_2_, Ser-C*H*α)), 4.21 (t, *J* = 6.8 Hz, 1H, FmocC*H*), 4.17 (dd, *J* = 10.7, 4.1 Hz, 1H, Ser-C*H*_2_β), 4.10 (d, *J* = 6.9 Hz, 2H, H6ab), 3.98 – 3.73 (m, 3H), 2.11 (s, 3H, H2, H5, Ser-C*H*_2_β), 2.03 (s, 1H), 2.01 (s, 3H), 1.98 (s, 3H) (2.03 – 1.98 Ac), 1.46 (s, 9H, tBu).

***^13^C-NMR*** (100.6 MHz, CDCl_3_, gHSQC, gHMBC), *δ (ppm)*: 170.61, 170.38, 168.80, 156.25, 143.97, 141.52, 127.97, 127.36, 127.34, 125.37, 120.20 (127.97 – 120.20 Fmoc), 100.95 (C1), 82.90 (*C*(CH_3_)_3_), 70.95 (C5), 70.12 (C3), 69.30 (Ser-*C*H_2_β), 66.99 (Fmoc*C*H_2_), 66.91 (C4), 61.68 (C6), 54.98 (Ser-*C*Hα), 51.66 (C2), 47.44 (Fmoc*C*H), 28.12 (tBu), 23.37, 21.25, 20.85 (23.37 – 20.80 Ac).

*N-9-Fluorenylmethoxycarbonyl-O-(2-N-acetamido-3,4,6-tri-O-acetyl-2-deoxy-β-D-galactopyranosyl)-L-serine* (**37**) *−* Compound **36** (1.51 g, 2.11 mmol) was dissolved in TFA/DCM 1:3 (30 mL), stirred at room temperature for 3 h. The solution was then concentrated and coevaporated with toluene (30 mL×3), concentrated and purification by silica column chromatography (DCM-MeOH 95:5 to 92:8) gave **37**. Yield: 91% (1.26 g, 1.92 mmol). R*_f_* = 0.3 (DCM/MeOH 9:1). *HR-ESI-MS (pos), m/z*: 657.2275 ([M+H]^+^, calculated 657.2296), 679.2098 ([M+Na]^+^, calculated 679.2115).

***^1^H-NMR*** (500 MHz, DMSO-*d*_6,_ gCOSY, gHSQC, gHMBC), *δ (ppm)*: δ 7.89 (dt, *J* = 7.4, 1.0 Hz, 2H, Fmoc), 7.85 (d, *J* = 9.1 Hz, 1H, N*H*Ac), 7.76 – 7.71 (m, 2H, Fmoc), 7.42 (tt, *J* = 7.5, 1.5 Hz, 2H, Fmoc), 7.33 (tdd, *J* = 7.4, 6.0, 1.2 Hz, 2H, Fmoc), 7.20 (d, *J* = 8.4 Hz, 1H, SerN*H*), 5.22 (d, *J* = 3.5 Hz, 1H, H4), 4.98 (dd, *J* = 11.2, 3.4 Hz, 1H, H3), 4.58 (d, *J* = 8.4 Hz, 1H, H1), 4.30 (d, *J* = 7.6 Hz, 2H, FmocC*H*_2_), 4.22 (td, *J* = 6.8, 6.2, 3.6 Hz, 2H, FmocC*H*, Ser-C*H*α), 4.04 (s, 3H, H5, H6ab), 3.99 (dd, *J* = 10.4, 6.2 Hz, 1H, Ser-C*H*_2_β), 3.88 (dt, *J* = 11.2, 8.8 Hz, 1H, H2), 3.75 (dd, *J* = 10.4, 4.1 Hz, 1H, Ser-C*H*_2_β), 2.10 (s, 3H), 1.99 (s, 3H), 1.90 (s, 3H), 1.74 (s, 3H) (2.10 - 1.74 Ac).

***^13^C-NMR*** (126 MHz, DMSO-*d*_6,_ gHSQC, gHMBC), *δ (ppm)*: 171.27, 169.96, 169.89, 169.75, 169.59, 155.93, 143.80, 143.70, 140.71, 140.70, 127.64, 127.08, 125.27, 125.21, 120.10 (127.64 – 120.10 Fmoc), 100.97 (C1), 70.31 (C3), 70.02 (C5), 68.41 (Ser-*C*H_2_β), 66.61 (C4), 65.87 (Fmoc*C*H_2_), 61.35 (C6), 53.95 (Ser-*C*Hα), 49.26 (C2), 46.60 (Fmoc*C*H), 22.73, 20.49, 20.44 (22.73 – 20.44 Ac)

*(2-N-Acetamido-2-deoxy-β-D-galactopyranosyl)-L-serine* (**38**) *−* Compound **37** (96 mg, 0.146 mmol) was dissolved in MeOH (10 mL), 1% NaOMe in MeOH was added to pH 11, stirred at room temperature for 48 h. After the reaction was finished, it was neutralized by 0.5 mL 1 M HCl, concentrated, and purification by silica column chromatography (DCM-MeOH-AcOH 10:1:1 to 1:1:1) gave **38**. Yield: 89% (40 mg, 0.130 mmol). R*_f_* = 0.3 (EtOAc/MeOH/H_2_O/AcOH 6:3:3:2). *HR-ESI-MS (pos), m/z*: 309.1283 ([M+H]^+^, calculated 309.1298), 331.1102 ([M+Na]^+^, calculated 331.1117).

***^1^H-NMR*** (500 MHz, D_2_O_,_ gCOSY, gHSQC, gHMBC), *δ (ppm)*: 4.45 (d, *J* = 8.5 Hz, 1H, H1), 4.11 (dd, *J* = 11.2, 7.5 Hz, 1H, Ser-C*H*_2_β), 4.06 (dd, *J* = 11.2, 3.9 Hz, 1H, Ser-C*H*_2_β), 3.92 – 3.85 (m, 3H, Ser-C*H*α, H4, H2), 3.77 (dd, *J* = 11.9, 8.1 Hz, 1H, H6a), 3.73 – 3.67 (m, 2H, H6b, H3), 3.64 (dd, *J* = 8.2, 3.9 Hz, 1H, H5), 2.01 (s, 3H, Ac).

***^13^C-NMR*** (126 MHz, D_2_O_,_ gHSQC, gHMBC), *δ (ppm)*: 175.15 (*C*OCH_3_), 171.51 (*C*OOH), 101.17 (C1), 75.17 (C5), 70.77 (C3), 67.75 (C4), 67.56 (Ser-*C*H_2_β), 61.09 (C6), 54.68 (Ser-*C*Hα), 52.16 (C2), 22.27 (Ac).

*(2-N-Acetamido-2-deoxy-β-D-glucopyranosyl)-L-threonine* (**40**) *−* Compound **39**(Chen et al., 2006) (103 mg, 0.154 mmol) was dissolved in MeOH (10 mL), 1% NaOMe in MeOH was added to pH 11, stirred at room temperature for 48 h. After the reaction was finished, it was neutralized by 0.5 mL 1 M HCl, concentrated, and purification by silica column chromatography (DCM-MeOH-AcOH 10:1:1 to 1:1:1) gave **40**. Yield: 70% (36 mg, 0.139 mmol). R*_f_* = 0.3 (EtOAc/MeOH/H_2_O/AcOH 6:3:3:2). *HR-ESI-MS (pos), m/z*: 345.1259 ([M+Na]^+^, calculated 345.1274).

***^1^H-NMR*** (500 MHz, D_2_O_,_ gCOSY, gHSQC, gHMBC), *δ (ppm)*: 4.39 (d, *J* = 8.4 Hz, 1H, H1), 4.14 (p, *J* = 6.3 Hz, 1H, Thr-C*H*β), 3.76 (dd, *J* = 12.3, 1.9 Hz, 1H, H6a), 3.60 (dd, *J* = 12.3, 5.1 Hz, 1H, H6b), 3.55 (dd, *J* = 10.4, 8.4 Hz, 1H, H2), 3.47 (d, *J* = 5.6 Hz, 1H, Thr-C*H*α), 3.39 (dd, *J* = 10.4, 8.0 Hz, 1H, H3), 3.33 – 3.25 (m, 2H, H2, H5), 1.91 (s, 3H, Ac), 1.15 (d, *J* = 6.5 Hz, 3H, Thr-C*H*_3_).

***^13^C-NMR*** (126 MHz, D_2_O, gHSQC, gHMBC), *δ (ppm)*: 174.84 (*C*OCH_3_), 172.05 (*C*OOH), 99.56 (C1), 75.67 (C5), 74.07 (Thr-*C*Hβ), 73.45 (C3), 69.62 (C4), 60.53 (C6), 59.22 (Thr-*C*Hα), 55.29 (C2), 22.01 (Ac), 17.29 (Thr-*C*H_3_).

*(2-N-Acetamido-2-deoxy-β-D-glucopyranosyl)-L-serine* (**42**) *−* Compound **41** (Chen et al. 2006) (105 mg, 0.146 mmol) was dissolved in 10 mL pH 10 NaOMe/MeOH, stirred at room temperature for 24 h. The solution was first neutralized by 150 AcOH and then concentrated to dryness. Pd/C (96 mg) was mixed with the intermediate product followed by addition of 6.4 mL 15:1 MeOH/AcOH, flushed with Ar and kept under H_2_ for overnight. After the reaction was finished, the catalyst was removed by filtration and the residue was concentrated, purification by silica column chromatography (DCM-MeOH-AcOH 10:1:1 to 1:1:1) gave **42**. Yield: 69% (31 mg, 0.100 mmol). R*_f_* = 0.3 (EtOAc/MeOH/H_2_O/AcOH 6:3:3:2). *HR-ESI-MS (pos), m/z*: 309.1295 ([M+H]^+^, calculated 309.1298), 331.1112 ([M+Na]^+^, calculated 331.1117).

***^1^H-NMR*** (500 MHz, D_2_O_,_ gCOSY, gHSQC, gHMBC), *δ (ppm)*: 4.41 (d, *J* = 8.4 Hz, 1H, H1), 4.01 (dd, *J* = 11.1, 7.3 Hz, 1H, Ser-C*H*_2_β), 3.94 (dd, *J* = 11.2, 3.8 Hz, 1H, Ser-C*H*_2_β), 3.83 – 3.76 (m, 2H, H6a, Ser-C*H*α), 3.64 – 3.56 (m, 2H, H6b, H2), 3.40 (dt, *J* = 9.9, 4.1 Hz, 1H, H3), 3.33 – 3.28 (m, 2H, H4, H5), 1.91 (s, 3H, Ac).

***^13^C-NMR*** (126 MHz, D_2_O_,_ gHSQC, gHMBC), *δ (ppm)*: 174.89 (*C*OCH_3_), 171.35 (*C*OOH), 100.73 (C1), 75.76 (C5), 73.58 (C4), 69.61 (C3), 67.67 (Ser-*C*H_2_β), 60.46 (C6), 55.22 (C2), 54.57 (Ser-*C*Hα), 22.09 (Ac).

*(2-N-Acetamido-2-deoxy-α-D-galactopyranosyl)-L-threonine* (**44**) *−* Compound **43** (Liebe and Kunz, 1997; Paulsen and Hölck, 1982) (105 mg, 0.157 mmol) was dissolved in MeOH (10 mL), 1% NaOMe in MeOH was added to pH 11, stirred at room temperature for 48 h. After the reaction was finished, it was neutralized by 0.5 mL 1 M HCl, concentrated, and purification by silica column chromatography (DCM-MeOH-AcOH 10:1:1 to 1:1:1) gave **44**. Yield: 87% (44 mg, 0.136 mmol). R*_f_* = 0.3 (EtOAc/MeOH/H_2_O/AcOH 6:3:3:2). *HR-ESI-MS (pos), m/z*: 345.1260 ([M+Na]^+^, calculated 345.1274).

***^1^H-NMR*** (500 MHz, D_2_O_,_ gCOSY, gHSQC, gHMBC), *δ (ppm)*: 4.94 (d, *J* = 3.8 Hz, 1H, H1), 4.45 (qd, *J* = 6.7, 2.0 Hz, 1H, Thr-C*H*β), 4.09 (dd, *J* = 11.1, 3.8 Hz, 1H, H2), 4.02 (t, *J* = 6.2 Hz, 1H, H5), 3.97 (d, *J* = 3.2 Hz, 1H, H4), 3.88 (dd, *J* = 11.2, 3.1 Hz, 1H, H3), 3.81 (d, *J* = 1.9 Hz, 1H, Thr-C*H*α), 3.76 – 3.68 (m, 2H, H6ab), 2.03 (s, 3H, Ac), 1.40 (d, *J* = 6.7 Hz, 3H, Thr-C*H*_3_).

***^13^C-NMR*** (126 MHz, D_2_O, gHSQC, gHMBC), *δ (ppm)*: 174.65 (*C*OCH_3_), 171.39 (*C*OOH), 99.17 (C1), 74.30 (Thr-*C*Hβ), 71.48 (C5), 68.48 (C4), 67.41 (C3), 61.32 (C6), 58.84 (Thr-*C*Hα), 49.73 (C2), 22.23 (Ac), 18.28 (Thr-*C*H_3_).

*(2-N-Acetamido-2-deoxy-α-D-galactopyranosyl)-L-serine* (**46**) *−* Compound **45** (Liebe and Kunz, 1997; Paulsen and Hölck, 1982) (96 mg, 0.146 mmol) was dissolved in MeOH (10 mL), 1% NaOMe in MeOH was added to pH 11, stirred at room temperature for 48 h. After the reaction was finished, it was neutralized by 0.5 mL 1 M HCl, concentrated, and purification by silica column chromatography (DCM-MeOH-AcOH 10:1:1 to 1:1:1) gave **46**. Yield: 51% (23 mg, 0.075 mmol). R*_f_* = 0.3 (EtOAc/MeOH/H_2_O/AcOH 6:3:3:2). *HR-ESI-MS (pos), m/z*: 309.1296 ([M+H]^+^, calculated 309.1298), 331.1112 ([M+Na]^+^, calculated 331.1117).

***^1^H-NMR*** (500 MHz, D_2_O_,_ gCOSY, gHSQC, gHMBC), *δ (ppm)*: 4.76 (d, *J* = 3.8 Hz, 1H, H1), 4.03 (dd, *J* = 11.0, 3.8 Hz, 1H H2), 3.96 (dd, *J* = 11.1, 3.0 Hz, 1H, Ser-C*H*_2_β), 3.87 – 3.71 (m, 5H, H3, H4, H5, Ser-C*H*α, Ser-C*H*_2_β), 3.68 – 3.56 (m, 2H, H6ab), 1.92 (s, 3H, Ac).

***^13^C-NMR*** (126 MHz, D_2_O_,_ gHSQC, gHMBC), *δ (ppm)*: 176.86 (*C*OCH_3_), 174.59 (*C*OOH), 97.91 (C1), 71.33 (C5), 68.31 (C3), 67.36 (C4), 66.45 (Ser-*C*H_2_β), 61.20 (C6), 54.36 (Ser-*C*Hα), 49.54 (C2), 20.46 (Ac).

**Supplementary References**

Adams EL, Rice PJ, Graves B, Ensley HE, Yu H, Brown GD, Gordon S, Monteiro MA, Papp-Szabo E, Lowman DW, et al. 2008. Differential High-Affinity Interaction of Dectin-1 with Natural or Synthetic Glucans Is Dependent upon Primary Structure and Is Influenced by Polymer Chain Length and Side-Chain Branching. *J Pharmacol Exp Ther* 325(1):115–123.

Bartels MF, Winterhalter PR, Yu J, Liu Y, Lommel M, Möhrlen F, Hu H, Feizi T, Westerlind U, Ruppert T, Strahl S. 2016. Protein O-mannosylation in the murine brain: Occurrence of Mono-O-Mannosyl glycans and identification of new substrates. *PLoS One* 11:e0166119.

Boraston AB, Warren RAJ, Kilburn DG. 2001. β-1,3-glucan binding by a thermostable carbohydrate-binding module from Thermotoga maritima. *Biochemistry* 40:14679–14685.

Capicciotti CJ, Mancini RS, Turner TR, Koyama T, Alteen MG, Doshi M, Inada T, Acker JP, Ben RN. 2016. O-Aryl-Glycoside Ice Recrystallization Inhibitors as Novel Cryoprotectants: A Structure-Function Study. *ACS Omega* 1:656–662.

Chai W, Stoll MS, Galustian C, Lawson AM, Feizi T. 2003. Neoglycolipid technology: Deciphering information content of glycome. *Methods Enzymol* 362:160–195.

Chen YX, Du JT, Zhou LX, Liu XH, Zhao YF, Nakanishi H, Li YM. 2006. Alternative O-GlcNAcylation/O-Phosphorylation of Ser16 Induce Different Conformational Disturbances to the N Terminus of Murine Estrogen Receptor β. *Chem Biol* 13:937–944.

Comer FI, Vosseller K, Wells L, Accavitti MA, Hart GW. 2001. Characterization of a mouse monoclonal antibody specific for O-linked N-acetylglucosamine*. Anal Biochem* 293:169–177.

Gao C, Liu Y, Zhang H, Zhang Y, Fukuda MN, Palma AS, Kozak RP, Childs RA, Nonaka M, Li Z, et al. 2014. Carbohydrate sequence of the prostate cancer-associated antigen F77 assigned by a mucin O-glycome designer array. *J Biol Chem* 289:16462–16477.

Godula K, Bertozzi CR. 2012. Density variant glycan microarray for evaluating cross-linking of mucin-like glycoconjugates by lectins. *J Am Chem Soc* 134:15732–15742.

Henshaw JL, Bolam DN, Pires MR, Czjzek M, Henrissat B, Ferreira LMA, Fontes CMGA, Gilbert HJ. 2004. The Family 6 Carbohydrate Binding Module Cm CBM6-2 Contains Two Ligand-binding Sites with Distinct Specificities * *J Biol Chem* 279:21552–21559.

Kumar V, Giri SK, Venugopalan P, Kartha KPR. 2014. Synthesis of Cross-Linked Glycopeptides and Ureas by a Mechanochemical, Solvent-Free Reaction and Determination of Their Structural Properties by TEM and X-ray Crystallography. *Chempluschem* 79:1605–1613.

Kunz H. 1997. O- and N-Glycopeptides: Synthesis of selectively deprotected building blocks. In: Hanessian S, editor. Preparative Carbohydrate Chemistry. Marcel Dekker, New York. p 265–281.

Lee WS, Kim W, Kim KT, Chung SK. 2011. Mitochondrial affinity of guanidine-rich molecular transporters built on monosaccharide scaffolds: Stereochemistry and lipophilicity. *Bull Korean Chem Soc* 32:2286–2300.

Lefeber DJ, Kamerling JP, Vliegenthart JFG. 2001. Synthesis of Streptococcus pneumoniae Type 3 Neoglycoproteins Varying in Oligosaccharide Chain Length, Loading and Carrier Protein. *Chem – A Eur J* 7:4411–4421.

Li Z, Gao C, Zhang Y, Palma AS, Childs RA, Silva LM, Liu Y, Jiang X, Liu Y, Chai W, Feizi T. 2018. O-Glycome Beam Search Arrays for Carbohydrate Ligand Discovery. *Mol Cell Proteomics* 17:121–133.

Liebe B, Kunz H. 1997. Solid-Phase Synthesis of a Tumor-Associated Sialyl-TN Antigen Glycopeptide with a Partial Sequence of the “Tandem Repeat” of the MUC-1 Mucin. *Angew Chemie Int Ed English* 36:618–621.

Likhosherstov L.M., Novikova O.S., Yamskov I.A. PVE. 2012. Synthesis of N-glycyl-beta-glycopyranosylamines , human milk fucooligosaccharide derivatives. *Russ Chem Bull* 61:1816–1817.

Likhosherstov LM, Novikova OS, Malysheva NN, Piskarev VE. 2015. Synthesis of mono- and di-α-l-fucosylated 2-acetamido-2-deoxy-N-glycyl-β-d-glucopyranosylamines modeling N-glycoprotein carbohydrate-peptide bond region based on 2-acetamido-N-(N-tert-butyloxycarbonylglycyl)-2-deoxy-β-d-glucopyranosylamine. *Russ Chem Bull* 64:1445–1450.

Likhosherstov LM, Novikova OS, Sakharov AM, Nysenko ZN, Kolotyrkina NG, Piskarev VE. 2016. Synthesis of N-aminoacyl-β-glycopyranosylamines — derivatives of natural sialooligosaccharides. *Russ Chem Bull* 65:1617–1624.

Lin C-C, Hsu T-S, Lu K-C, Huang I-T. 2000. Synthesis of β-D-Glucopyranosyl(1→3)-1-thiol-β-glucosamine Disaccharide Derivative as Building Block for the Synthesis of Hyaluronic Acid. *J Chinese Chem Soc* 47:921–928.

Liu Y, Feizi T, Campanero-Rhodes MA, Childs RA, Zhang Y, Mulloy B, Evans PG, Osborn HMI, Otto D, Crocker PR, Chai W. 2007. Neoglycolipid Probes Prepared via Oxime Ligation for Microarray Analysis of Oligosaccharide-Protein Interactions. *Chem Biol* 14:847–859.

Liu Y, Ramelot TA, Huang P, Liu Y, Li Z, Feizi T, Zhong W, Wu F, Tan M, Kennedy MA, Jiang X. 2016. Glycan Specificity of P[19] Rotavirus and Comparison with Those of Related P Genotypes. *J Virol* 90:9983–9996.

Marcelo F, Garcia-Martin F, Matsushita T, Sardinha J, Coelho H, Oude-Vrielink A, Koller C, André S, Cabrita EJ, Gabius H-J, et al. 2014. Delineating Binding Modes of Gal/GalNAc and Structural Elements of the Molecular Recognition of Tumor-Associated Mucin Glycopeptides by the Human Macrophage Galactose-Type Lectin. *Chem – A Eur J* 20:16147–16155.

Michihata N, Kaneko Y, Kasai Y, Tanigawa K, Hirokane T, Higasa S, Yamada H. 2013. High-Yield Total Synthesis of (−)-Strictinin through Intramolecular Coupling of Gallates. *J Org Chem* 78:4319–4328.

Mohler DL, Shen G. 2006. The synthesis of tethered ligand dimers for PPARγ–RXR protein heterodimers. *Org Biomol Chem* 4:2082–2087.

Mortezai N, Behnken HN, Kurze AK, Ludewig P, Buck F, Meyer B, Wagener C. 2013. Tumor-associated Neu5Ac-Tn and Neu5Gc-Tn antigens bind to C-type lectin CLEC10A (CD301, MGL). *Glycobiology* 23:844–852.

Nishimura S-I, Kuzuhara H, Takiguchi Y, Shimahara K. 1989. Peracetylated chitobiose: Preparation by specific degradations of chitin, and chemical manipulations. *Carbohydr Res* 194:223–231.

Palma AS, Feizi T, Zhang Y, Stoll MS, Lawson AM, Díaz-Rodríguez E, Campanero-Rhodes MA, Costa J, Gordon S, Brown GD, Chai W. 2006. Ligands for the β-glucan receptor, dectin-1, assigned using “designer” microarrays of oligosaccharide probes (neoglycolipids) generated from glucan polysaccharides. *J Biol Chem* 281:5771–5779.

Palma AS, Liu Y, Childs RA, Herbert C, Wang D, Chai W, Feizi T. 2011. The human epithelial carcinoma antigen recognized by monoclonal antibody AE3 is expressed on a sulfoglycolipid in addition to neoplastic mucins. *Biochem Biophys Res Commun* 408:548–552.

Palma AS, Liu Y, Zhang H, Zhang Y, McCleary B V, Yu G, Huang Q, Guidolin LS, Ciocchini AE, Torosantucci A, et al. 2015. Unravelling glucan recognition systems by glucome microarrays using the designer approach and mass spectrometry. *Mol Cell Proteomics* 14:974–988.

Parkkinen J, Finne J. 1983. Isolation and structural characterization of five major sialyloligosaccharides and a sialylglycopeptide from normal human urine. *Eur J Biochem* 136:355–361.

Paulsen H, Hölck J-P. 1982. Synthesis of glycopeptide O-β-d-galactopyranosyl-(1→3)-O-(2-acetamido-2-desoxy-α-d-galactopyranosyl)-(1→3)-l-serin und -l-threonin. *Carbohydr Res* 109:89–107.

Pettenuzzo N, Brustolin L, Coltri E, Gambalunga A, Chiara F, Trevisan A, Biondi B, Nardon C, Fregona D. 2019. CuII and AuIII Complexes with Glycoconjugated Dithiocarbamato Ligands for Potential Applications in Targeted Chemotherapy. *ChemMedChem* 14:1162–1172.

Piskarev VE, Shuster AM, Gabibov AG, Rabinkov AG. 1990. A novel preparative method for the isolation of avidin and riboflavin-binding glycoprotein from chicken egg-white by the use of high-performance liquid chromatography. *Biochem J* 265:301–304.

Puri KD, Gopalakrishnan B, Surolia A. 1992. Carbohydrate binding specificity of the Tn-antigen binding lectin from Vicia villosa seeds (VVLB4). *FEBS Lett* 312:208–212.

Sanchez JF, Lescar J, Chazalet V, Audfray A, Gagnon J, Alvarez R, Breton C, Imberty A, Mitchell EP. 2006. Biochemical and structural analysis of Helix pomatia agglutinin: A hexameric lectin with a novel fold. *J Biol Chem* 281:20171–20180.

Šardzík R, Noble GT, Weissenborn MJ, Martin A, Webb SJ, Flitsch SL. 2010. Preparation of aminoethyl glycosides for glycoconjugation. *Beilstein J Org Chem* 6:699–703.

Takamiya R, Ohtsubo K, Takamatsu S. 2013. The interaction between Siglec-15 and tumor-associated sialyl-Tn antigen enhances TGF- β secretion from monocytes / macrophages through the DAP12 – Syk pathway. *Glycobiology* 23:178–187.

Tanaka H, Kawai T, Adachi Y, Hanashima S, Yamaguchi Y, Ohno N, Takahashi T. 2012. Synthesis of β(1,3) oligoglucans exhibiting a Dectin-1 binding affinity and their biological evaluation. *Bioorganic Med Chem* 20:3898–3914.

Varki A, Cummings RD, Aebi M, Packer NH, Seeberger PH, Esko JD, Stanley P, Hart G, Darvill A, Kinoshita T, et al. 2015. Symbol nomenclature for graphical representations of glycans. *Glycobiology* 25:1323–1324.

Vendele I, Willment JA, Silva LM, Palma AS, Chai W, Liu Y, Feizi T, Spyrou M, Stappers MHT, Brown GD, Gow NAR. 2020. Mannan detecting C-type lectin receptor probes recognise immune epitopes with diverse chemical, spatial and phylogenetic heterogeneity in fungal cell walls. *PLoS Pathog* 16:1–29.

Weishaupt MW, Hahm HS, Geissner A, Seeberger PH. 2017. Automated glycan assembly of branched β-(1,3)-glucans to identify antibody epitopes. *Chem Commun* 53:3591–3594.

Weishaupt MW, Matthies S, Seeberger PH. 2013. Automated solid-phase synthesis of a β-(1,3)-glucan dodecasaccharide. C*hem - A Eur J* 19:12497–12503.

Yarlagadda V, Konai MM, Manjunath GB, Ghosh C, Haldar J. 2015. Tackling vancomycin-resistant bacteria with “lipophilic-vancomycin-carbohydrate conjugates.” *J Antibiot* (Tokyo) 68:302–312.

Zhang P, Wang K, Zhang J, Li C, Guan H. 2015. Total Synthesis of Sulfated Glycosphingolipid SM1a, a Kind of Human Epithelial Carcinoma Antigen. *European J Org Chem* 2015:570–583.

Zhu T, Boons G-J. 1999. A Two-Directional and Highly Convergent Approach for the Synthesis of the Tumor-Associated Antigen Globo-H. *Angew Chemie Int Ed* 38:3495–3497.
